# Supplementary material for: Design, Synthesis and Bioactivity Evaluation of 4,6-Disubstituted Pyrido[3,2-d]pyrimidine Derivatives as Mnk and HDAC Inhibitors
Source: Molecules. 2020 Sep 21;25(18):4318. doi: 10.3390/molecules25184318 (PMC7571151; doi:10.3390/molecules25184318)
Supplement: Supplementary file 1 [file molecules-25-04318-s001.pdf]

molecules

Supplementary Materials

Design, Synthesis and Bioactivity Evaluation of 4,6-disubstituted pyrido[3,2-*d*]pyrimidine Derivatives as Mnk and HDAC inhibitors

Kun Xing, Jian Zhang, Yu Han, Tong Tong, Dan Liu* and Linxiang Zhao*

Key Laboratory of Structure-Based Drug Design & Discovery of Ministry of Education, Shenyang Pharmaceutical University, Shenyang 110016, China;

Correspondence: sammyld@163.com (D.L.); linxiang.zhao@vip.sina.com (L.Z.); Tel.: +024-4352-0221 (L.Z.)

1. Spectrum of target compounds

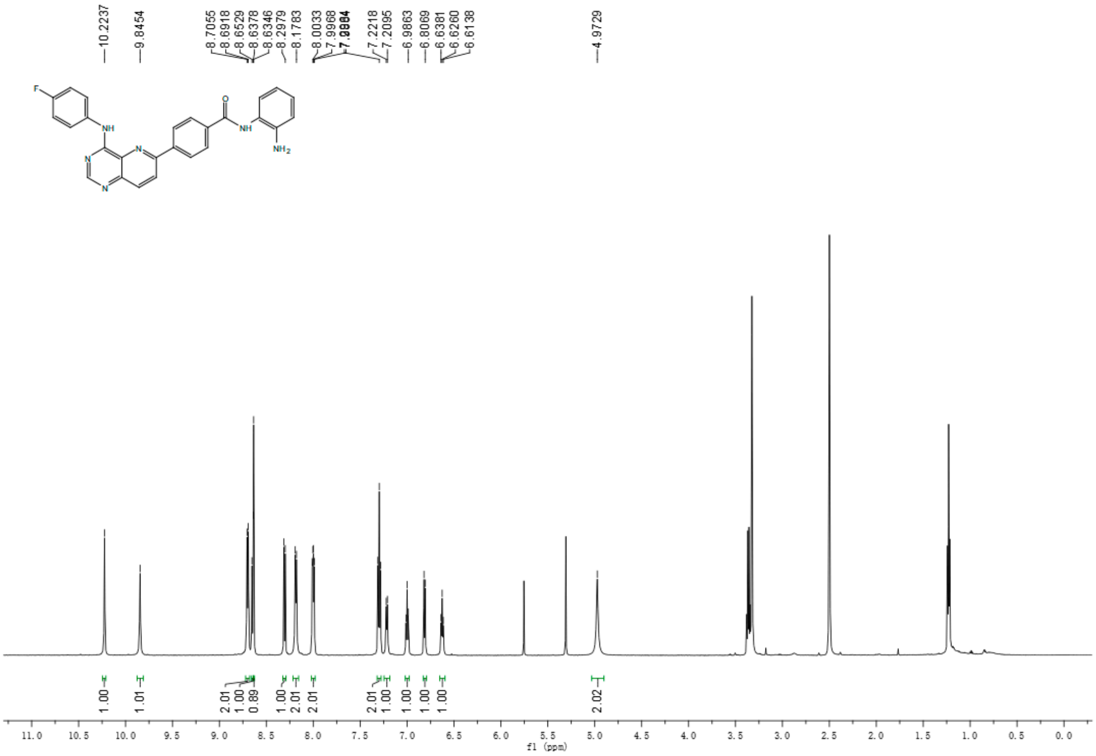

^{1}H-NMR spectra of compound A01

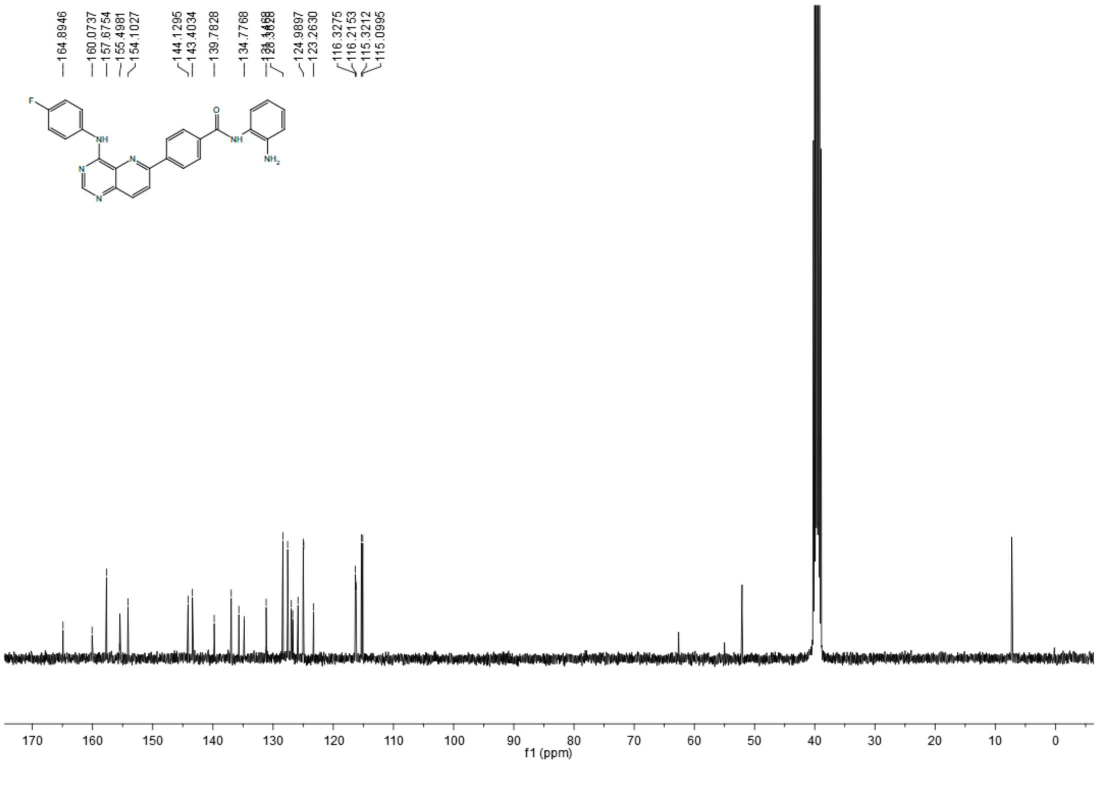

^{13}C-NMR spectra of compound A01

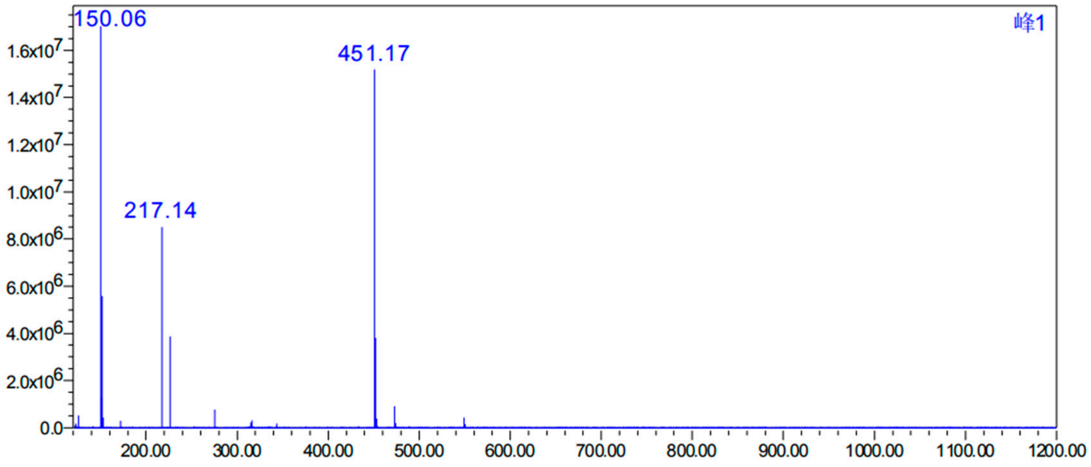

Ms spectra of compound A01

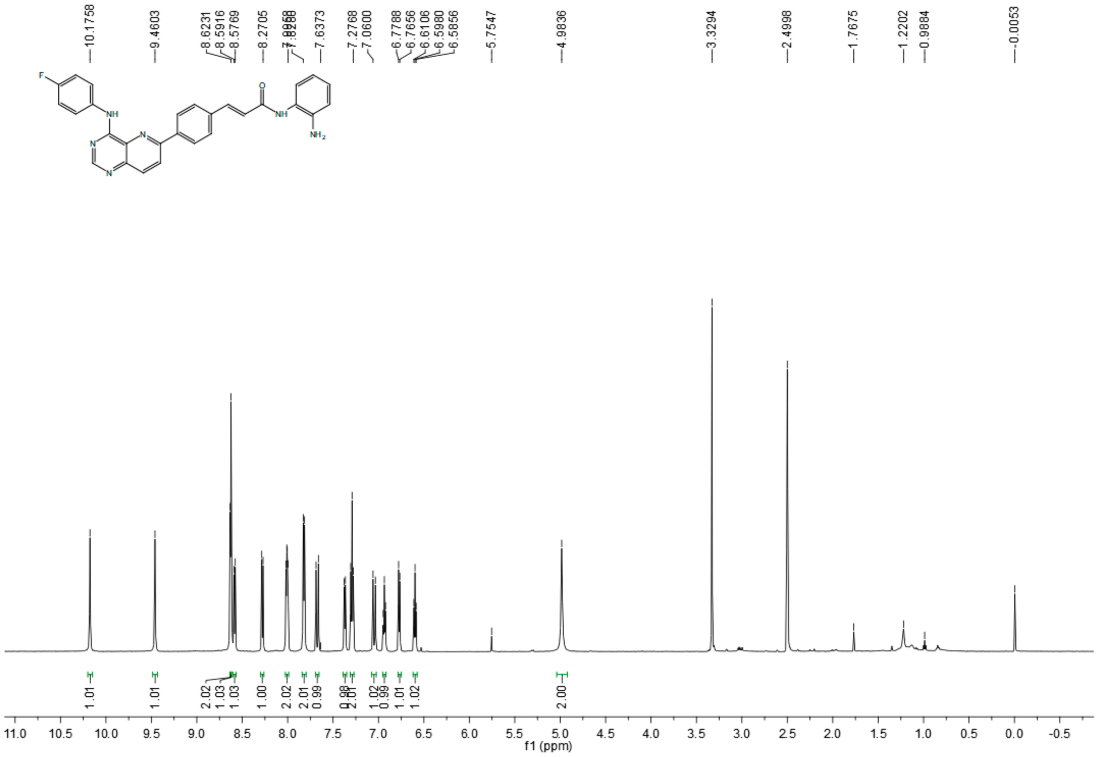

^{1}H-NMR spectra of compound A02

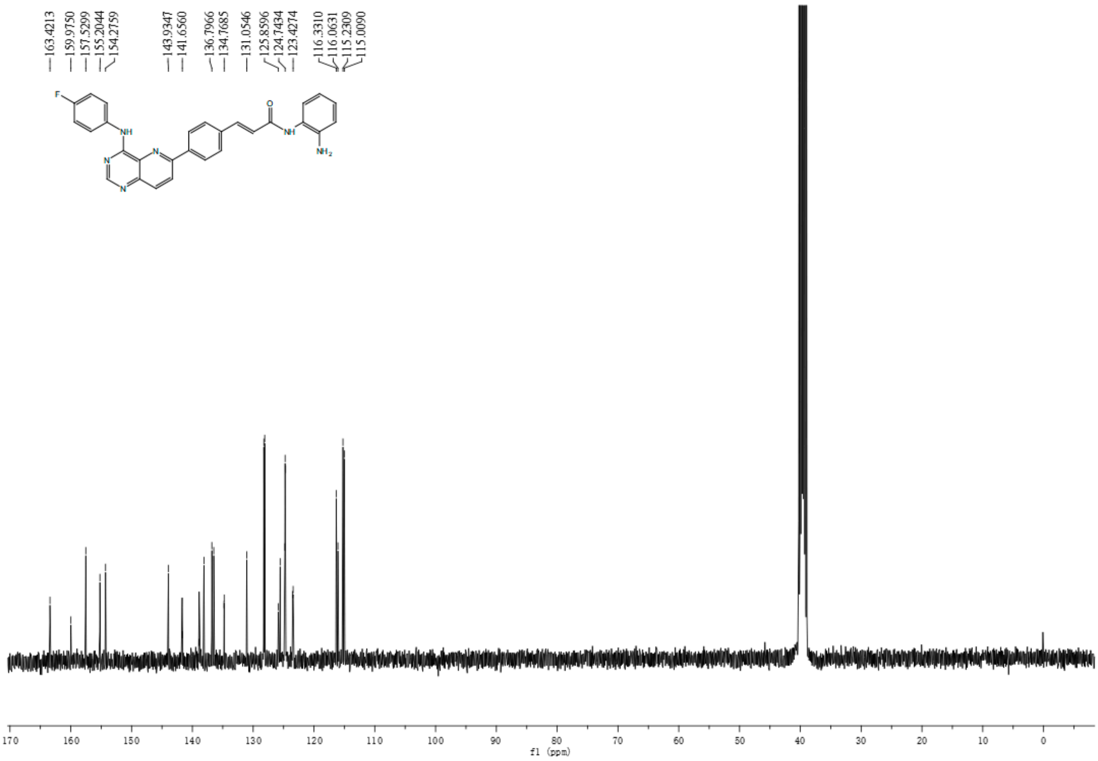

^{13}C-NMR spectra of compound A02

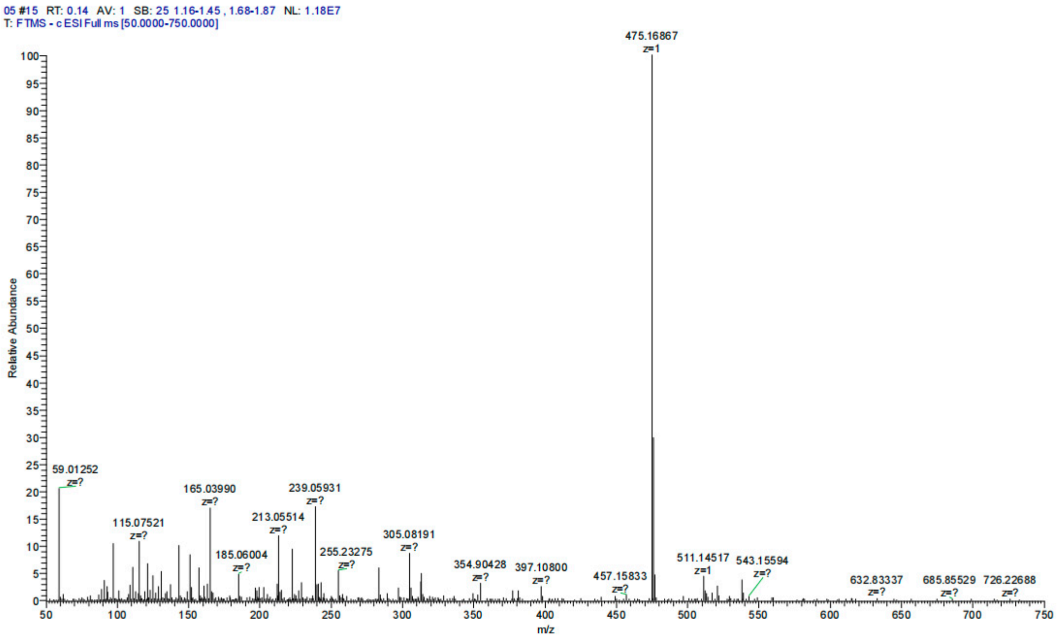

Ms spectra of compound A02

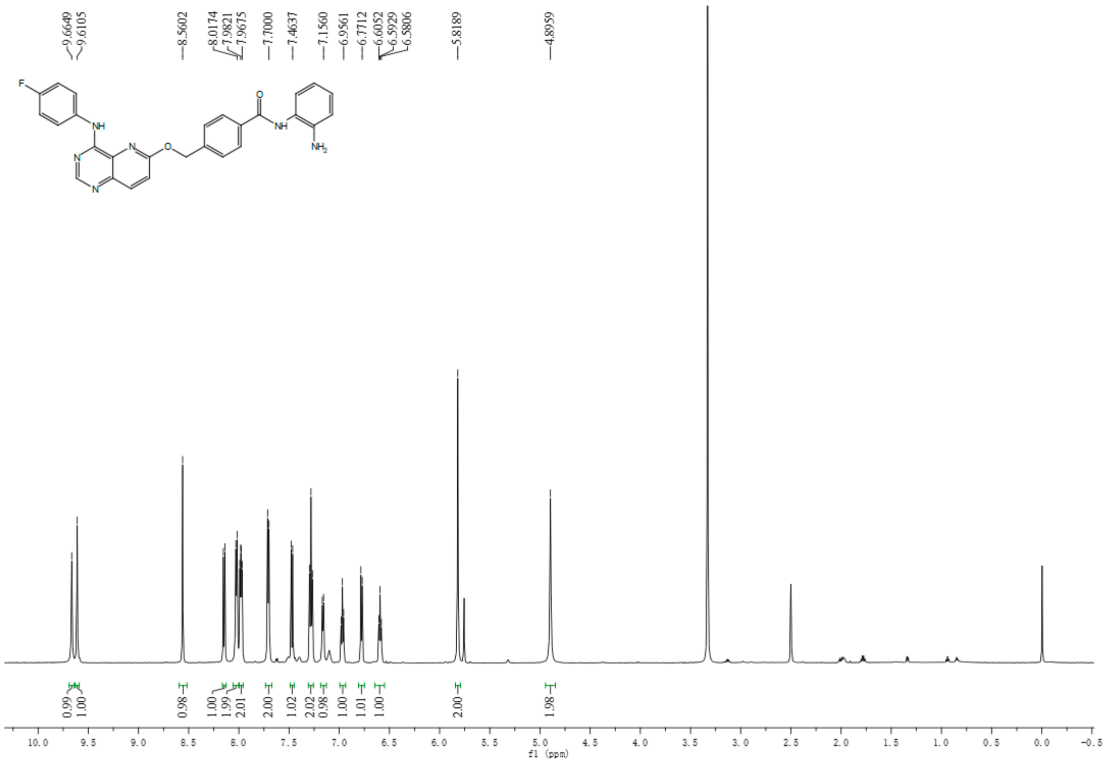

^{1}H-NMR spectra of compound A03

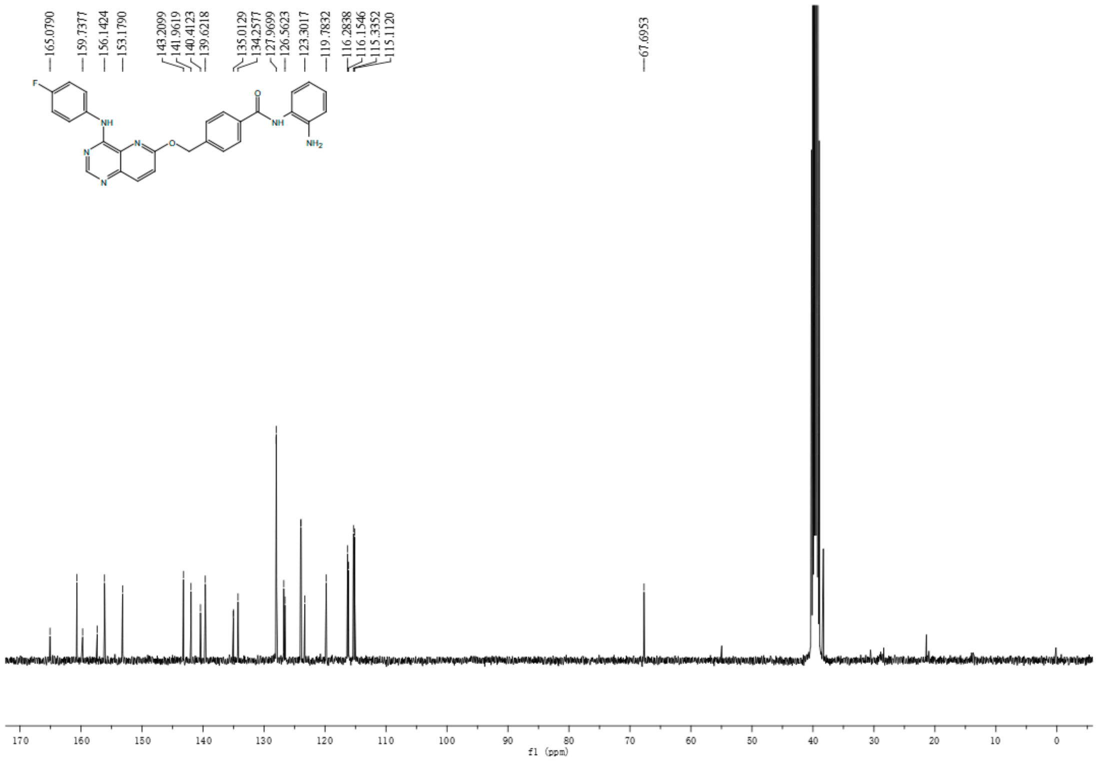

^{13}C-NMR spectra of compound A03

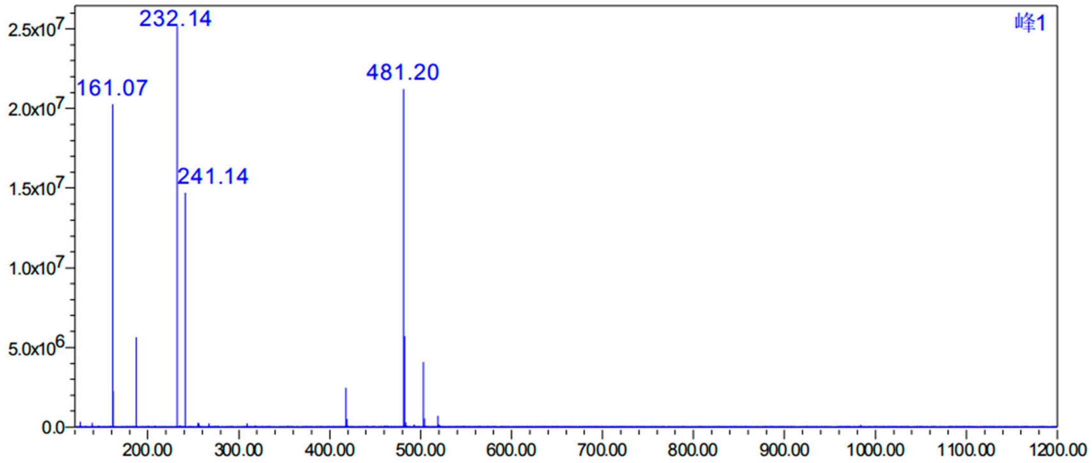

Ms spectra of compound A03

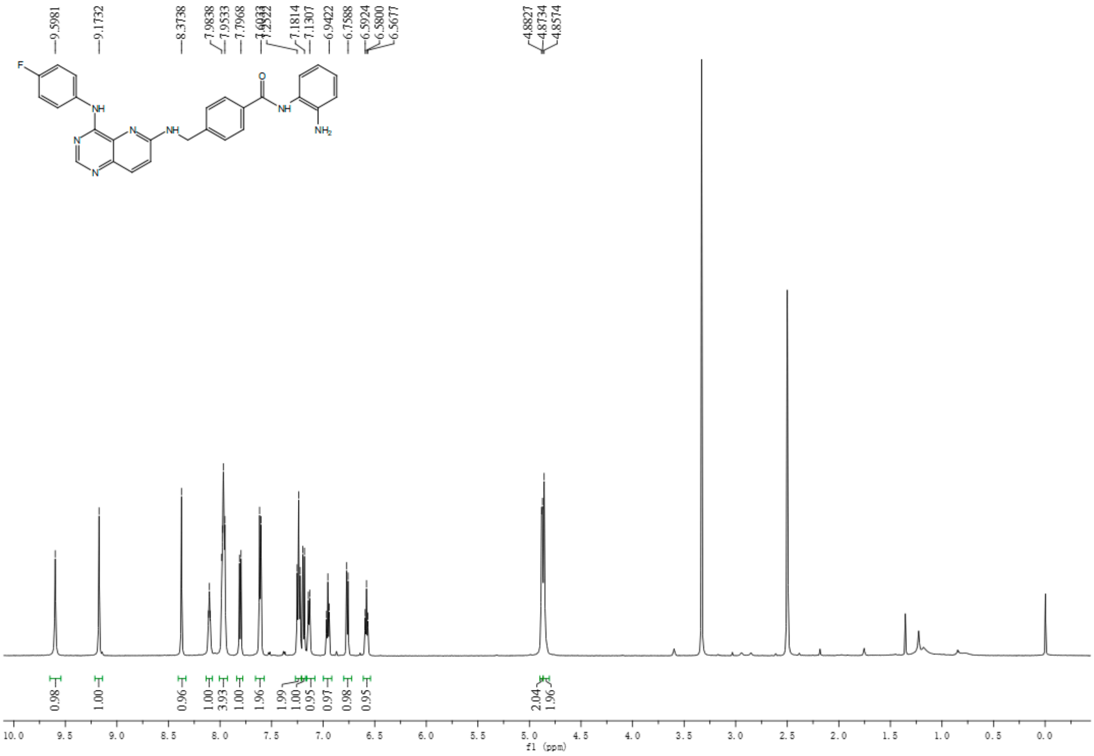

^{1}H-NMR spectra of compound A04

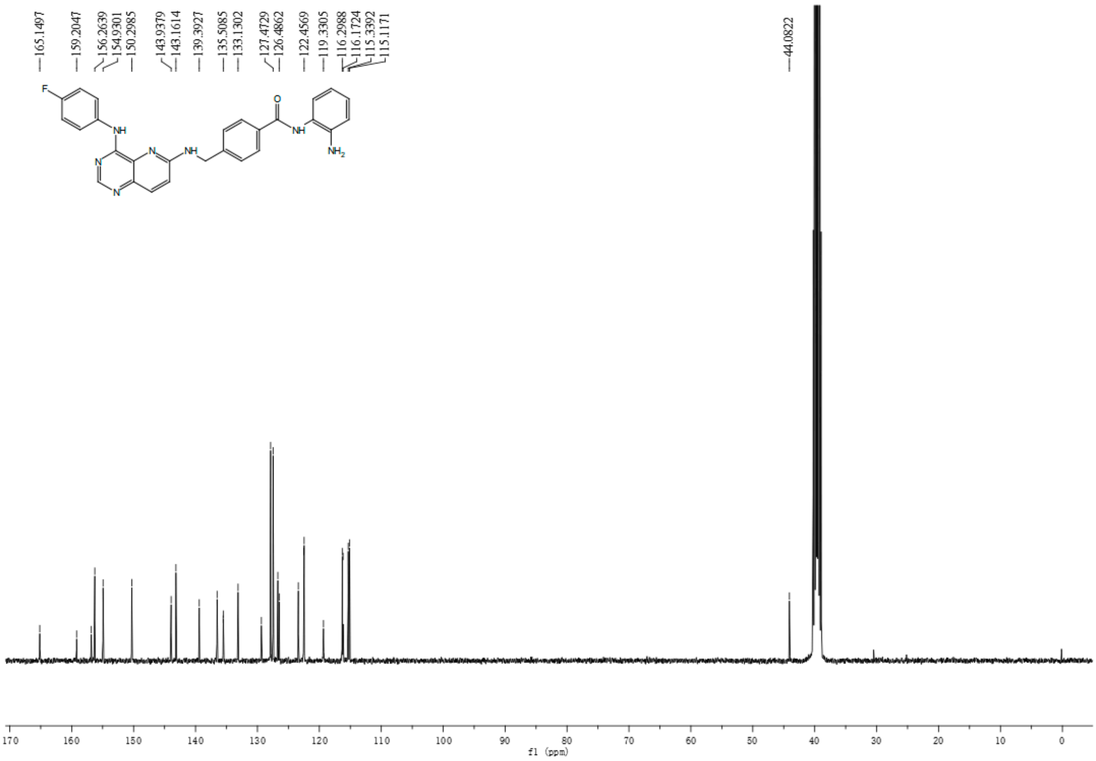

^{13}C-NMR spectra of compound A04

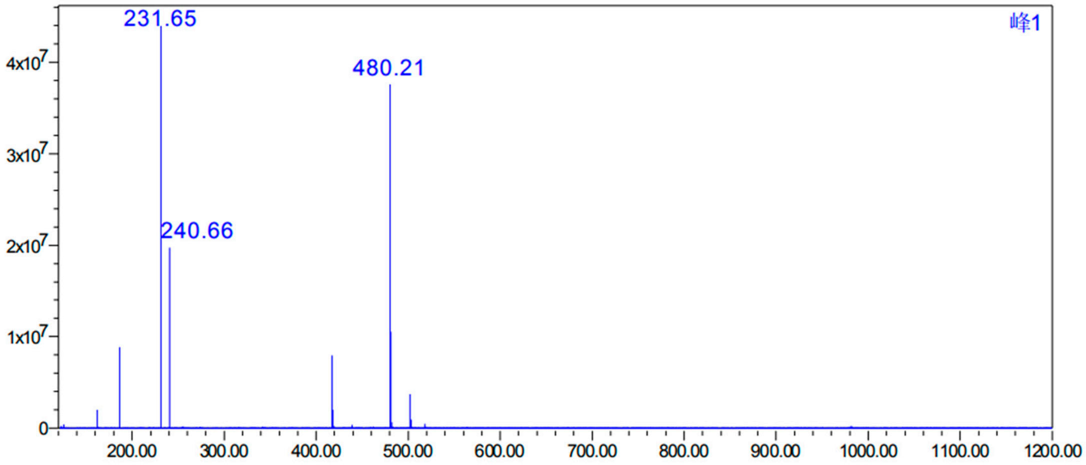

Ms spectra of compound A04

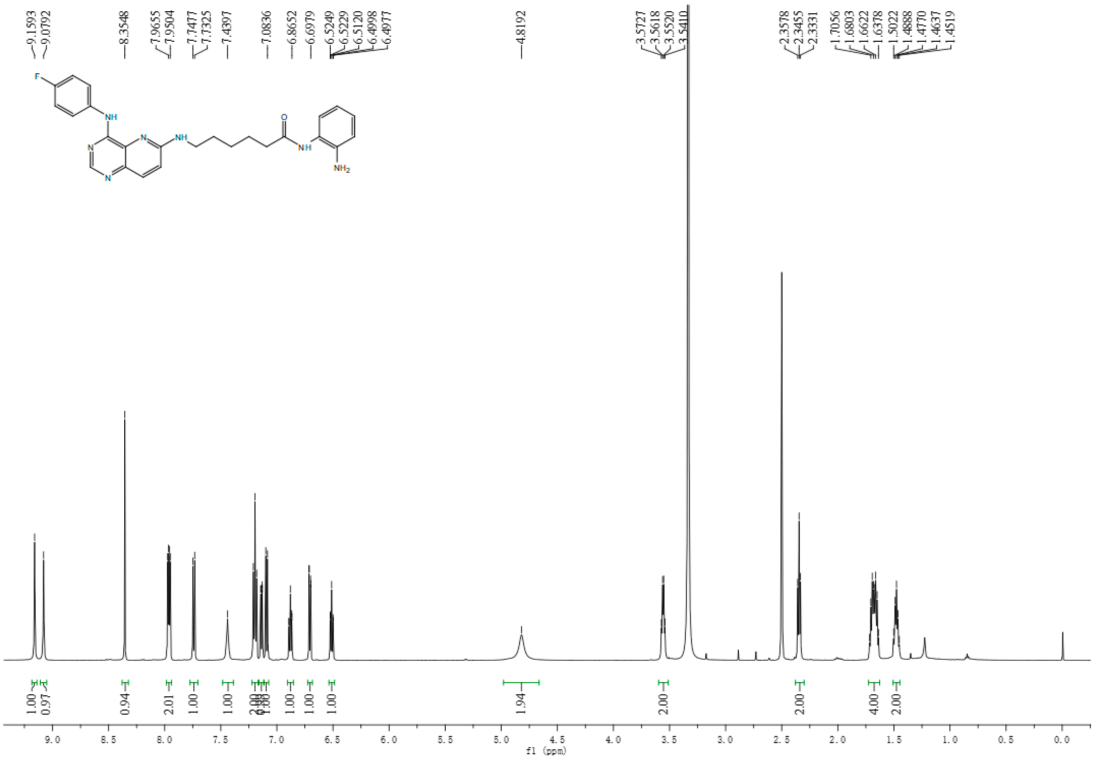

^{1}H-NMR spectra of compound A05

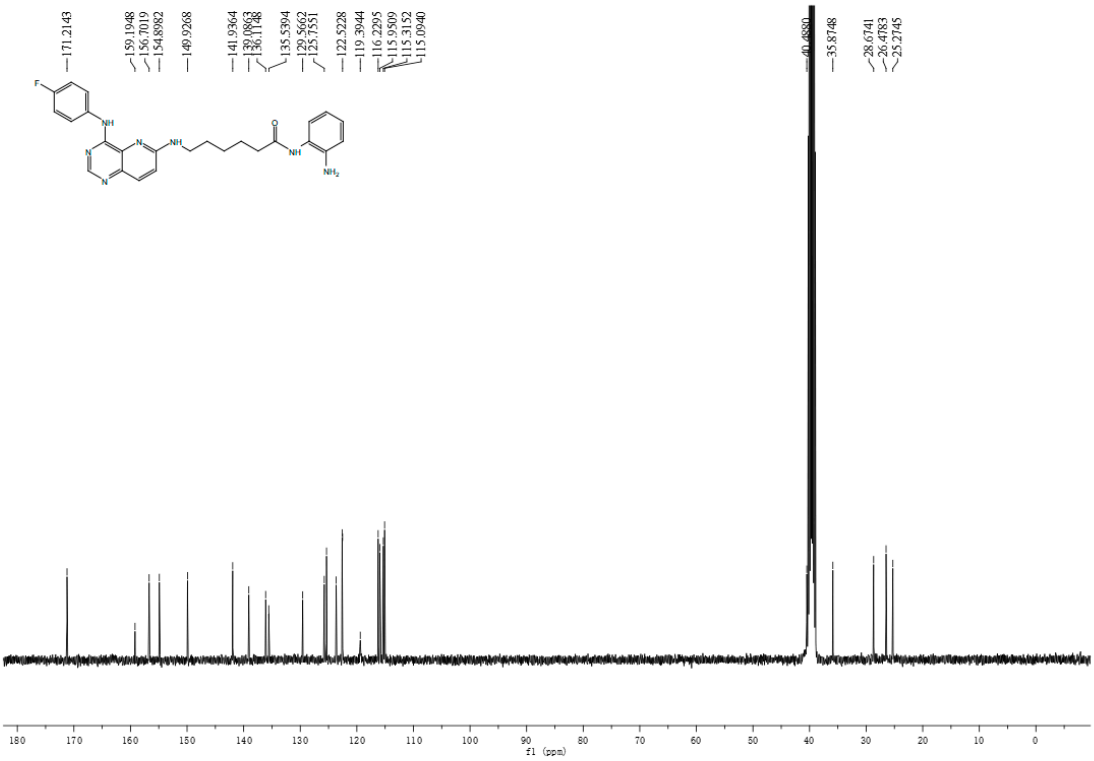

^{13}C-NMR spectra of compound A05

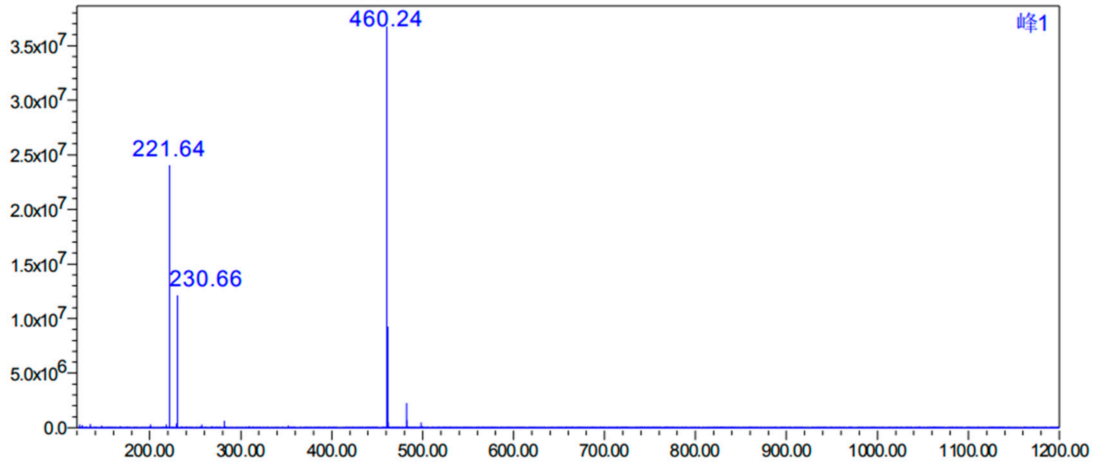

Ms spectra of compound A05

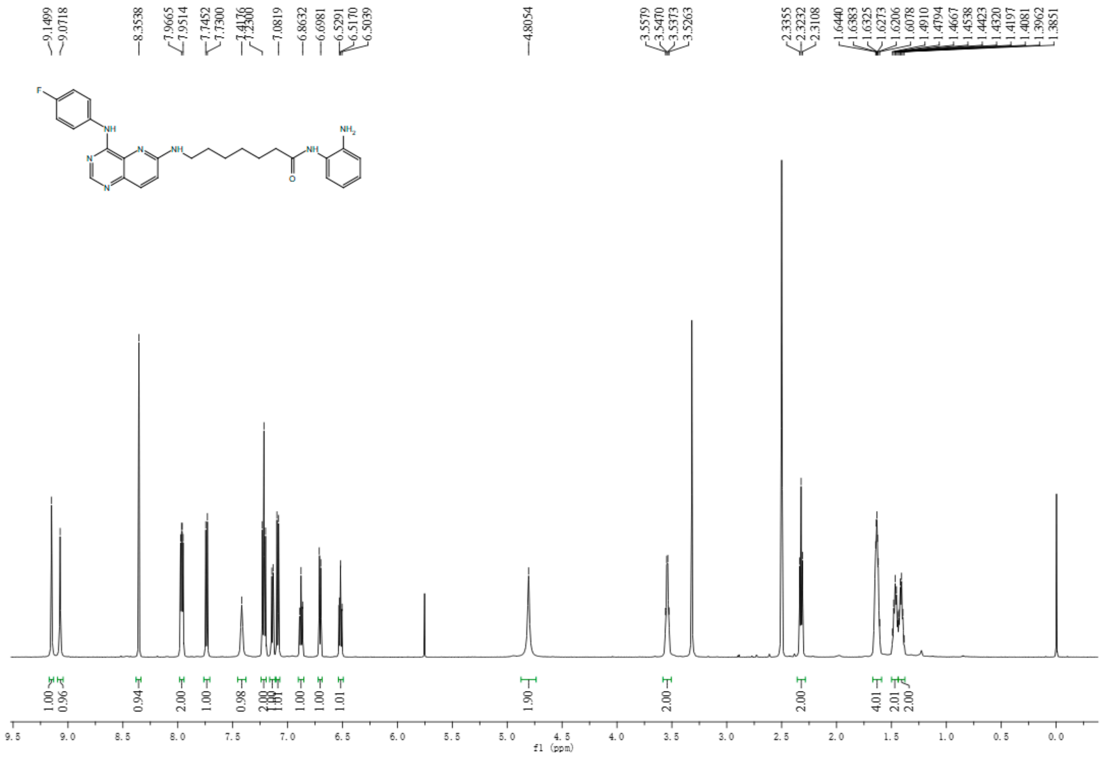

^{1}H-NMR spectra of compound A06

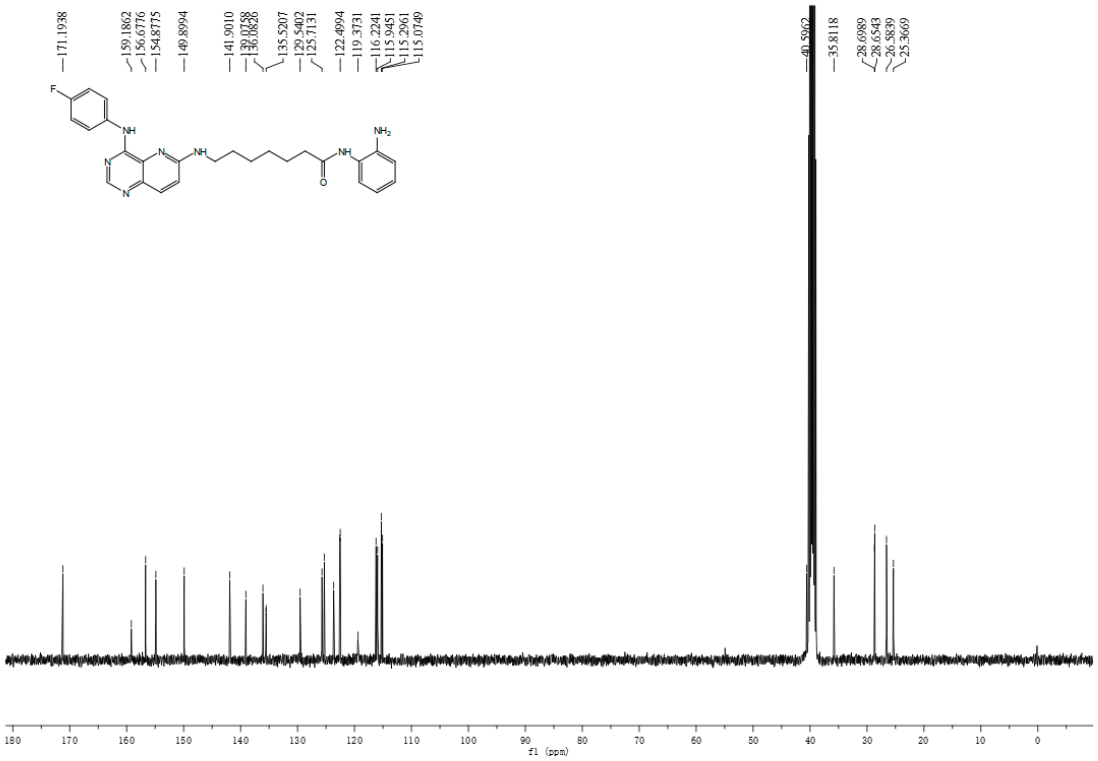

^{13}C-NMR spectra of compound A06

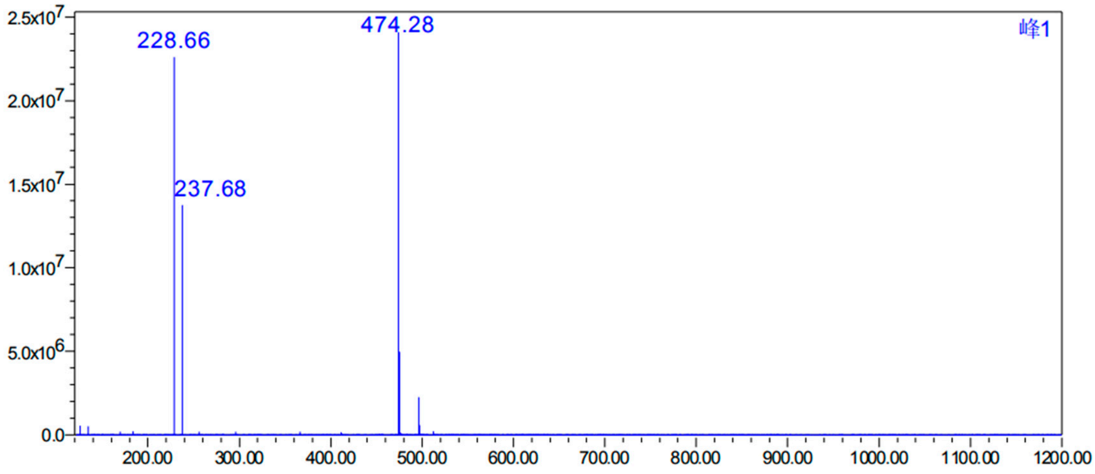

Ms spectra of compound A06

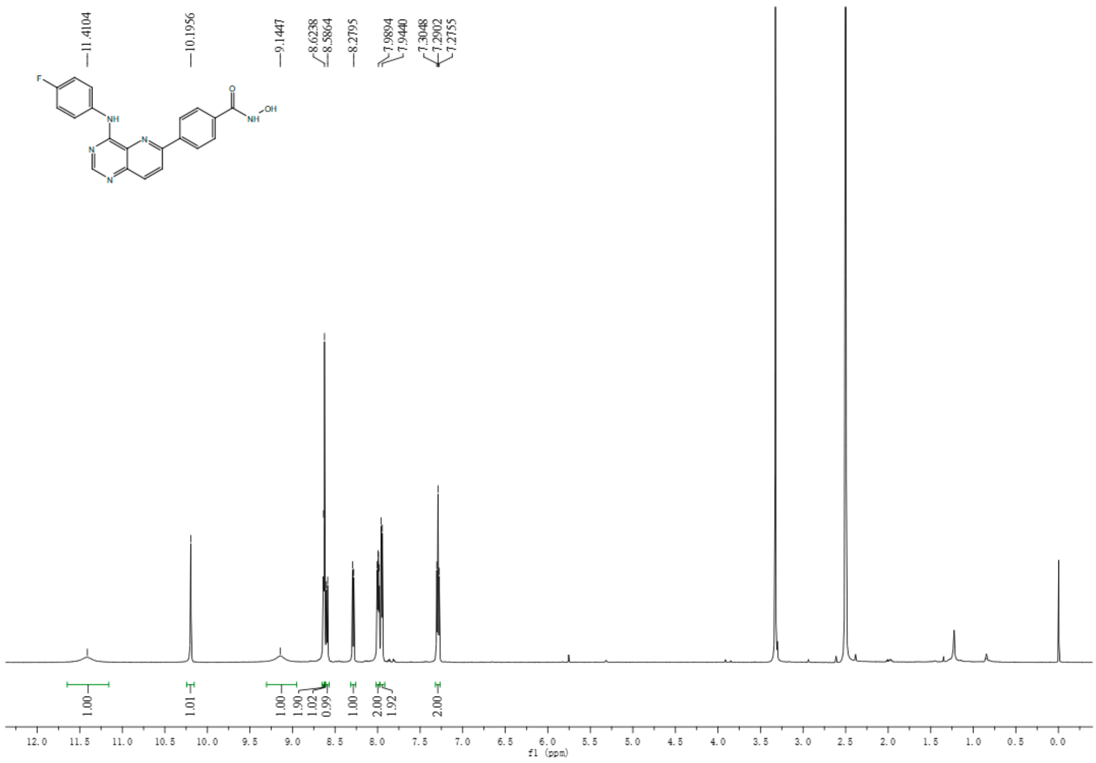

^{1}H-NMR spectra of compound A07

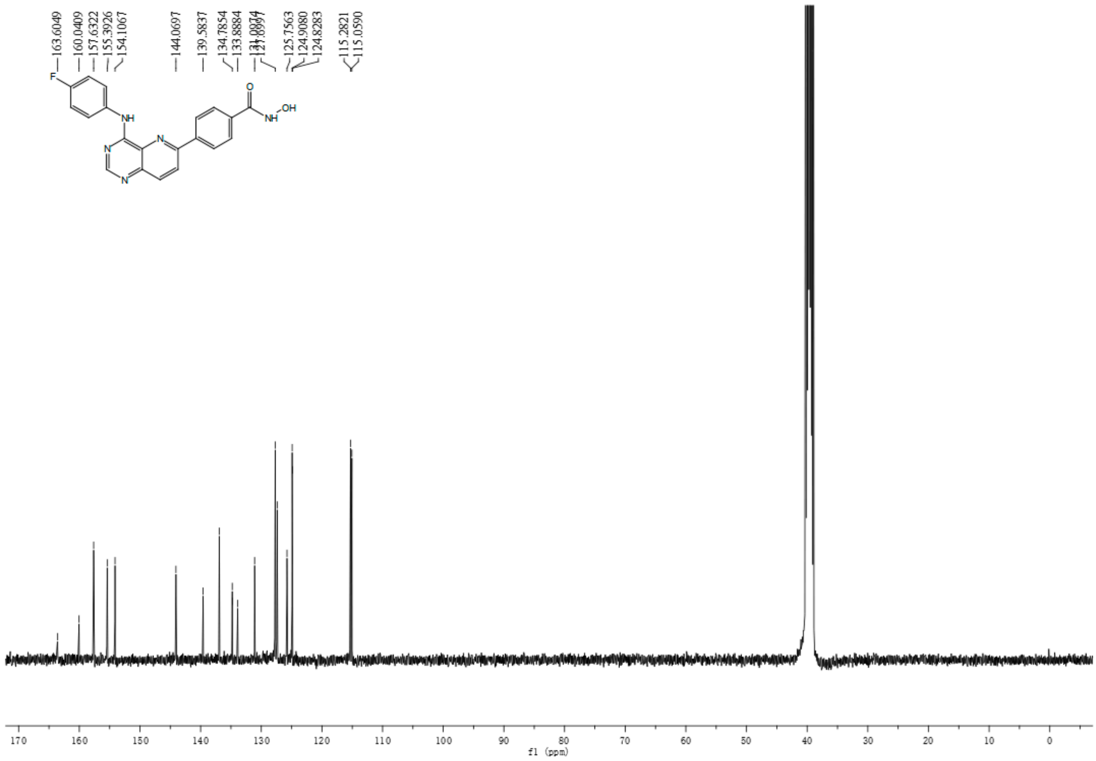

^{13}C-NMR spectra of compound A07

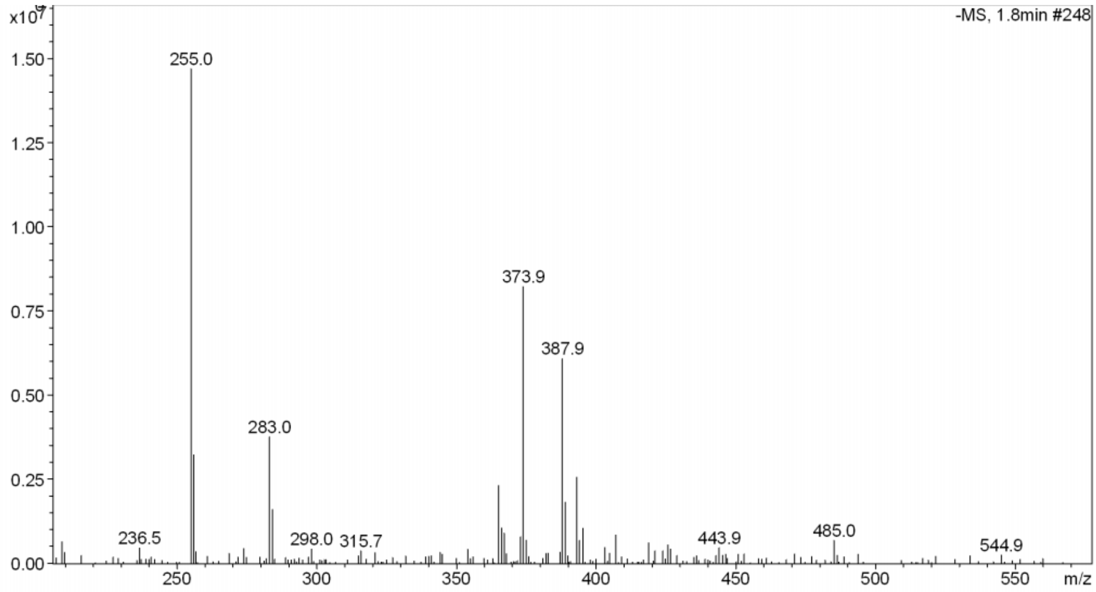

Ms spectra of compound A07

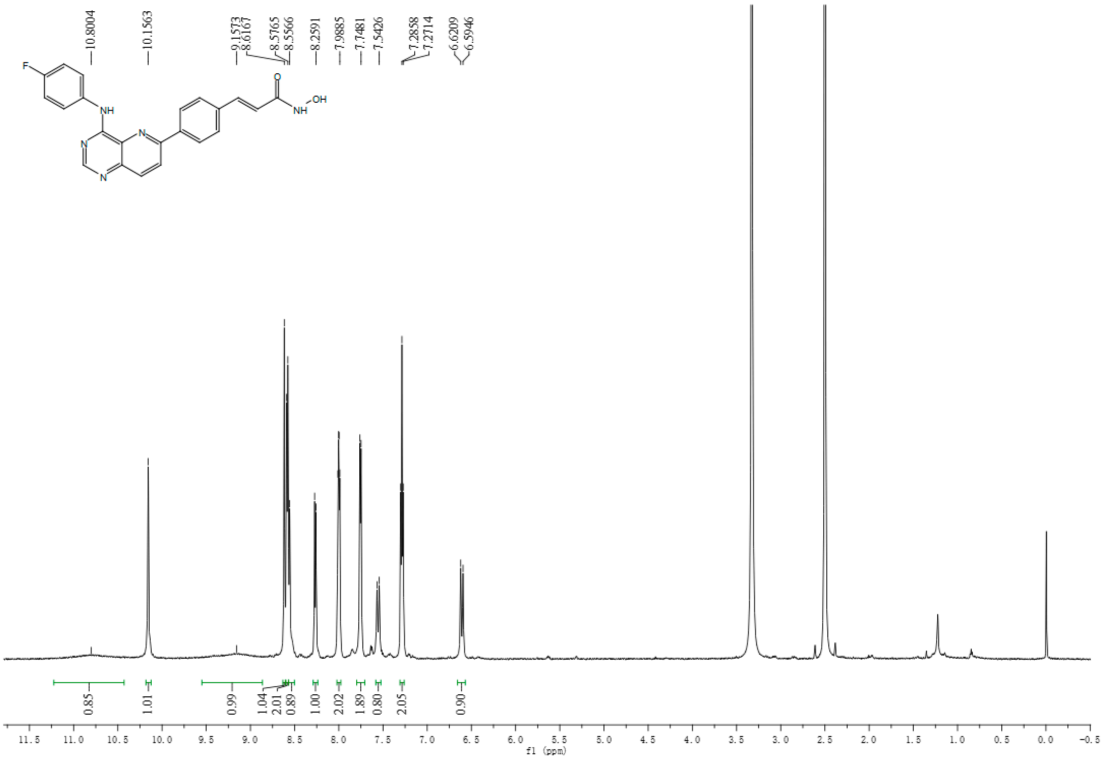

^{1}H-NMR spectra of compound A08

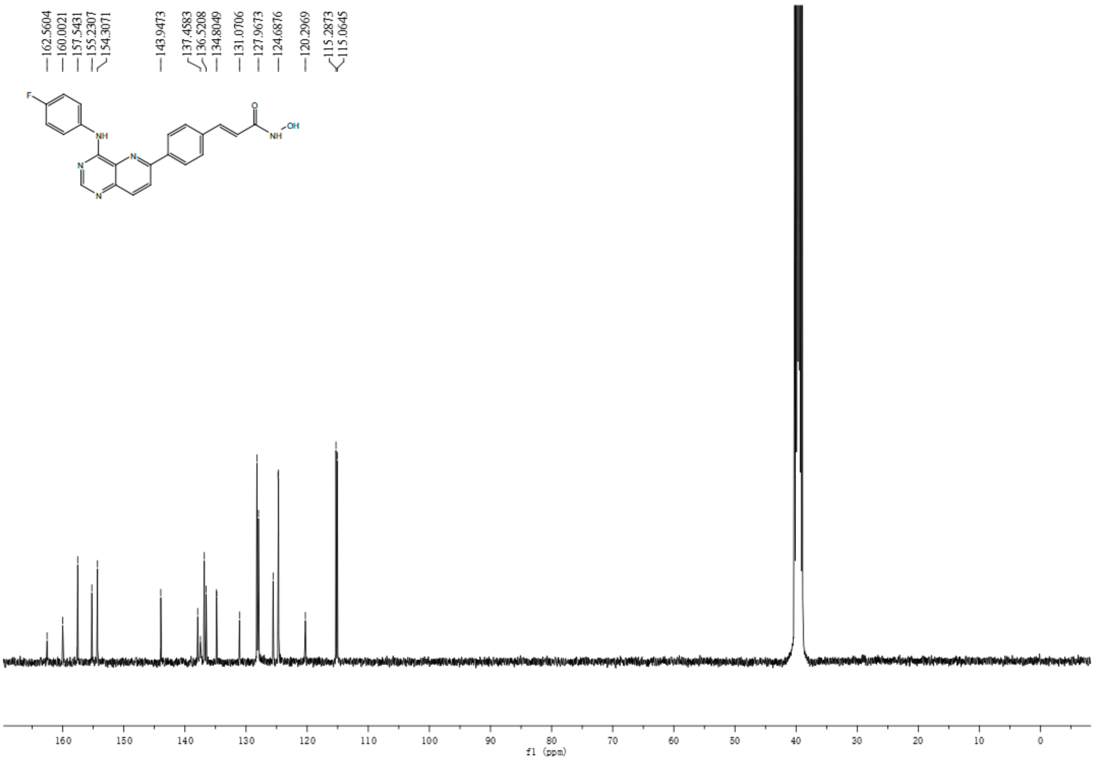

^{13}C-NMR spectra of compound A08

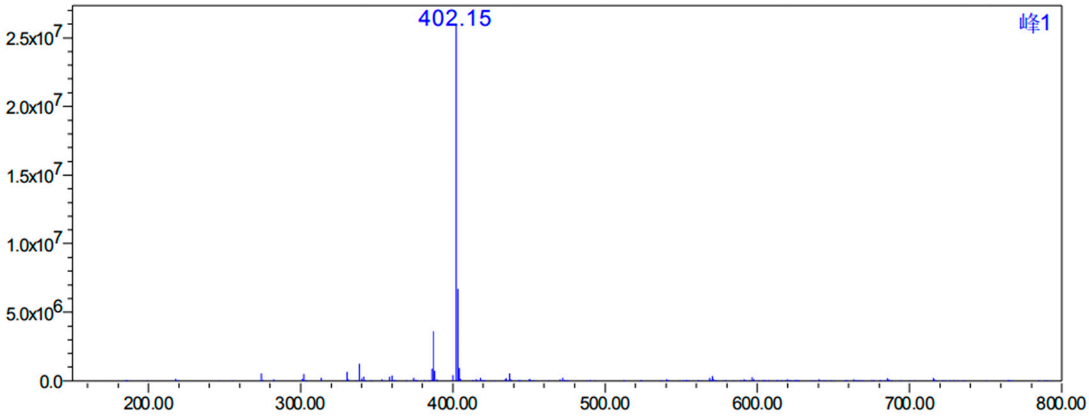

Ms spectra of compound A08

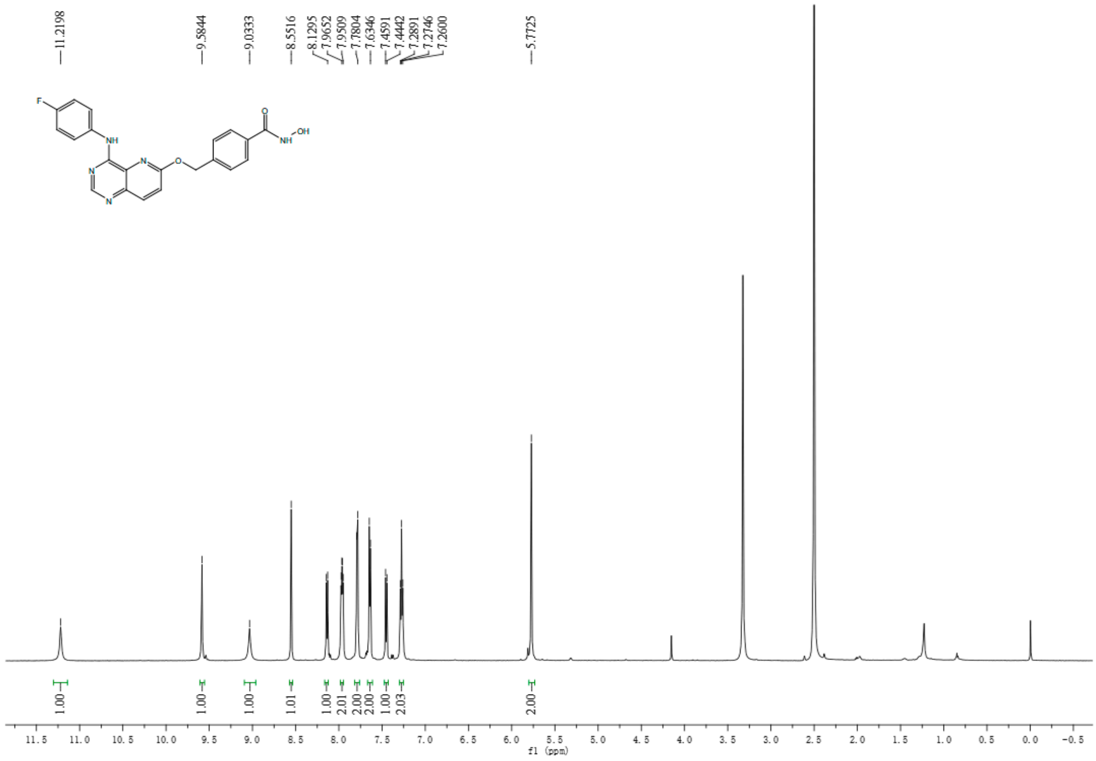

^{1}H-NMR spectra of compound A09

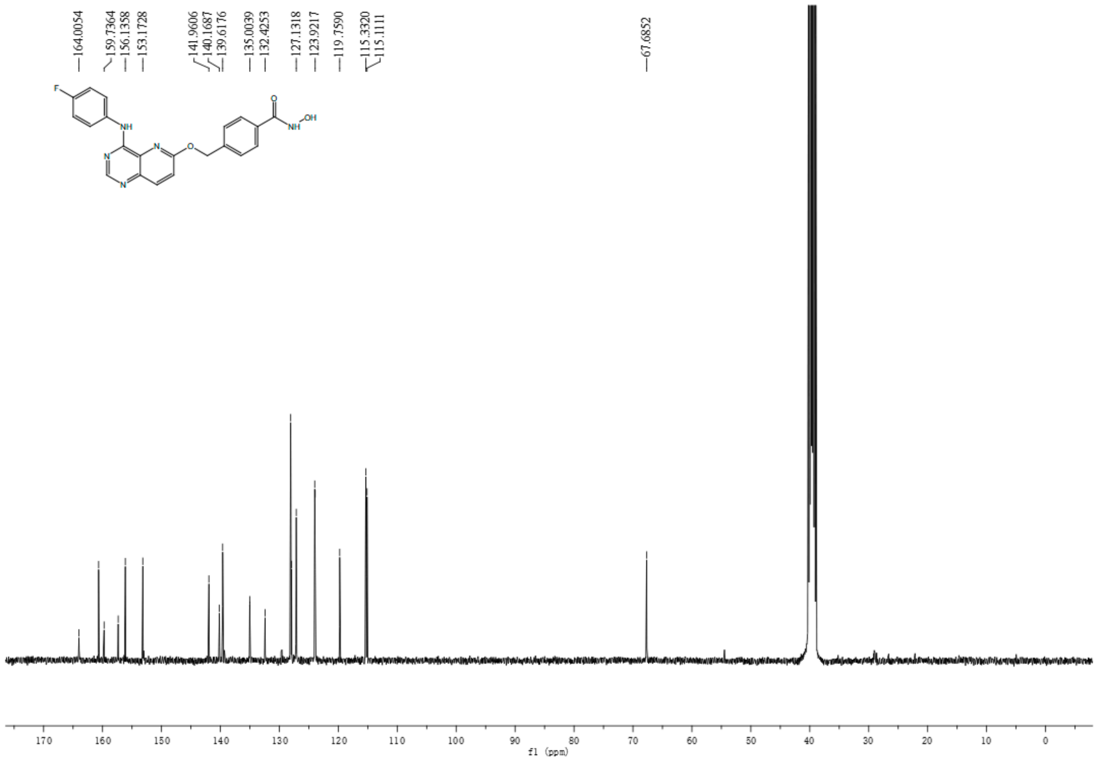

^{13}C-NMR spectra of compound A09

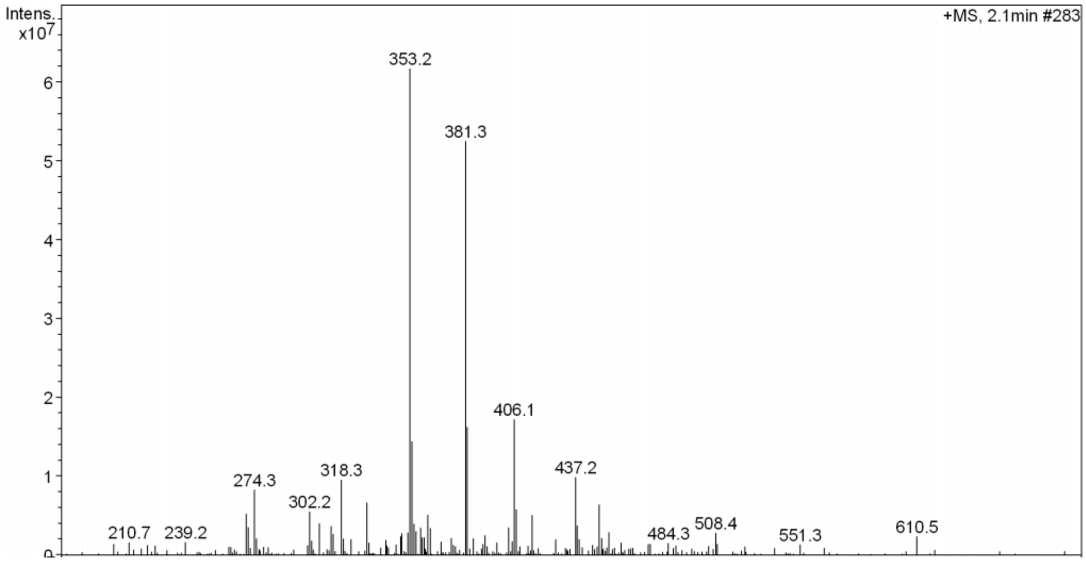

Ms spectra of compound A09

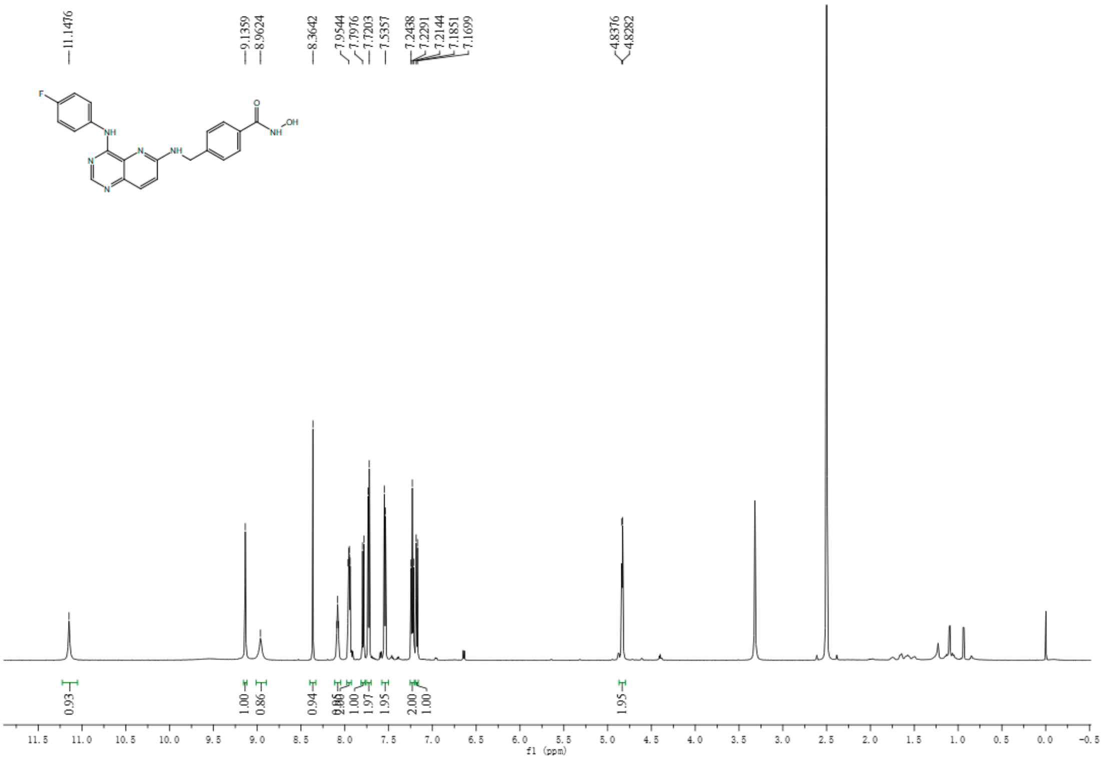

^{1}H-NMR spectra of compound A10

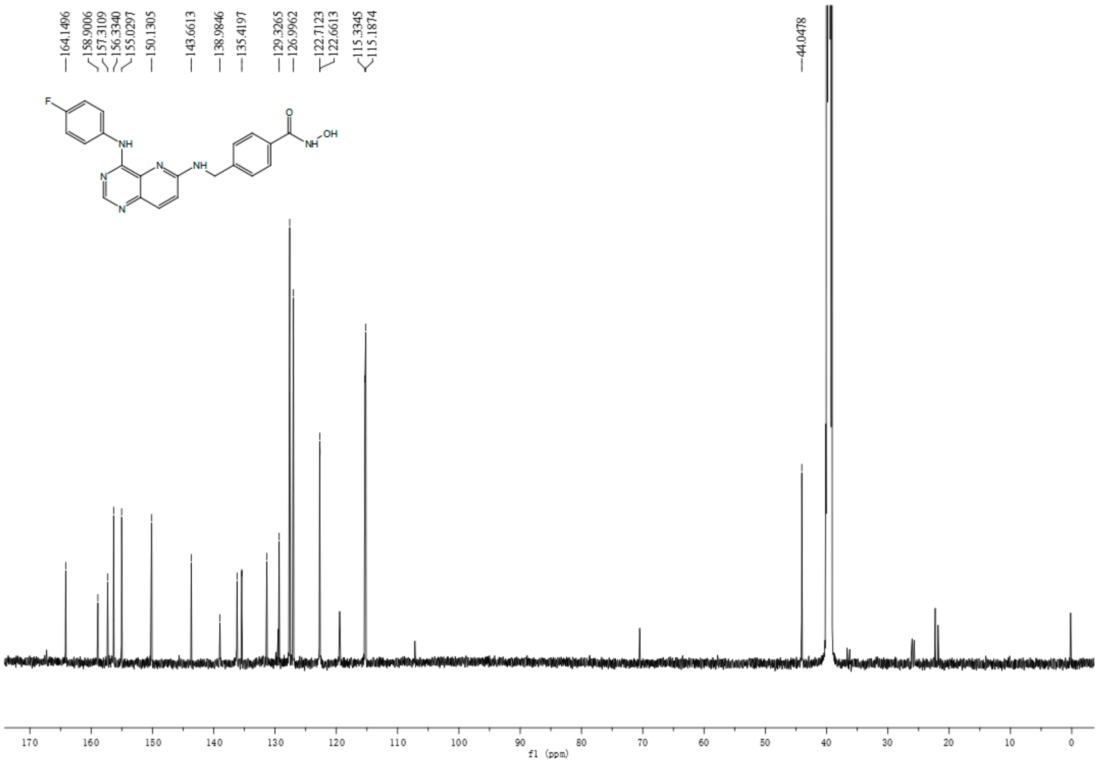

^{13}C-NMR spectra of compound A10

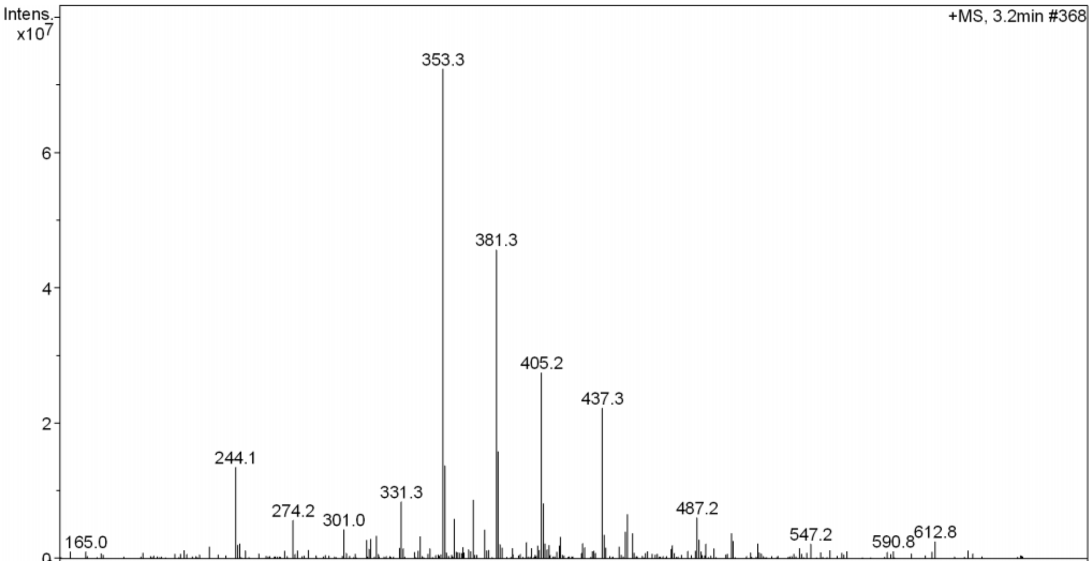

Ms spectra of compound A10

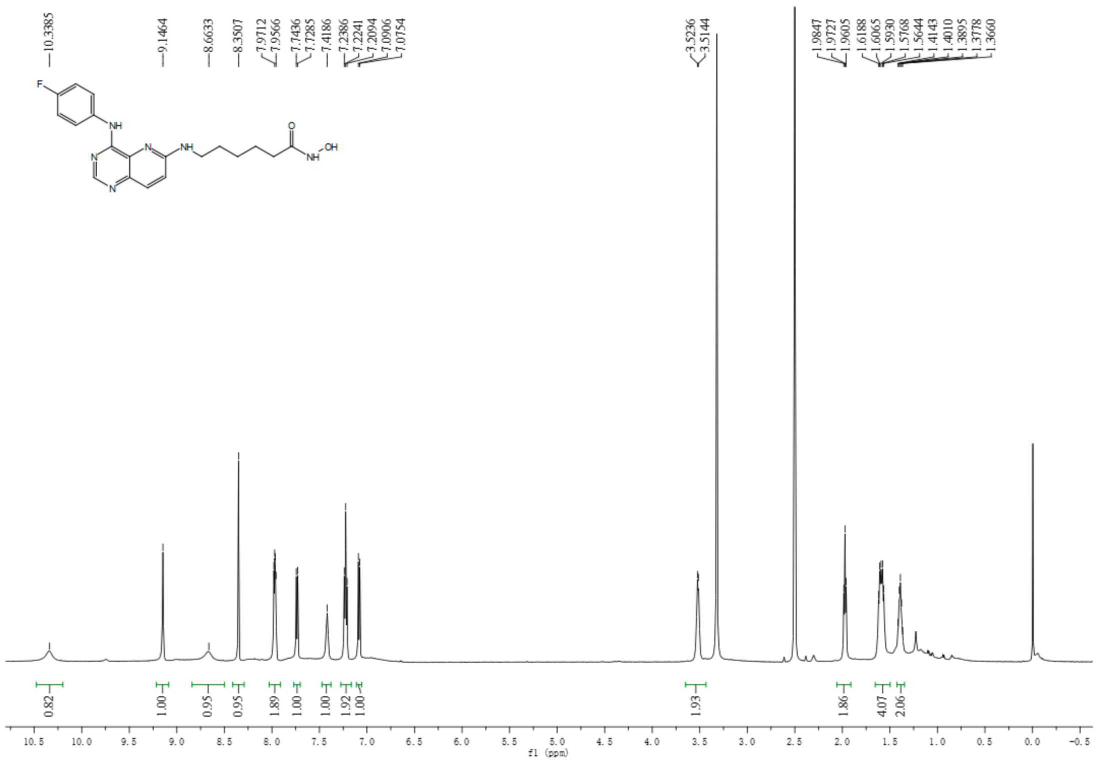

^{1}H-NMR spectra of compound A11

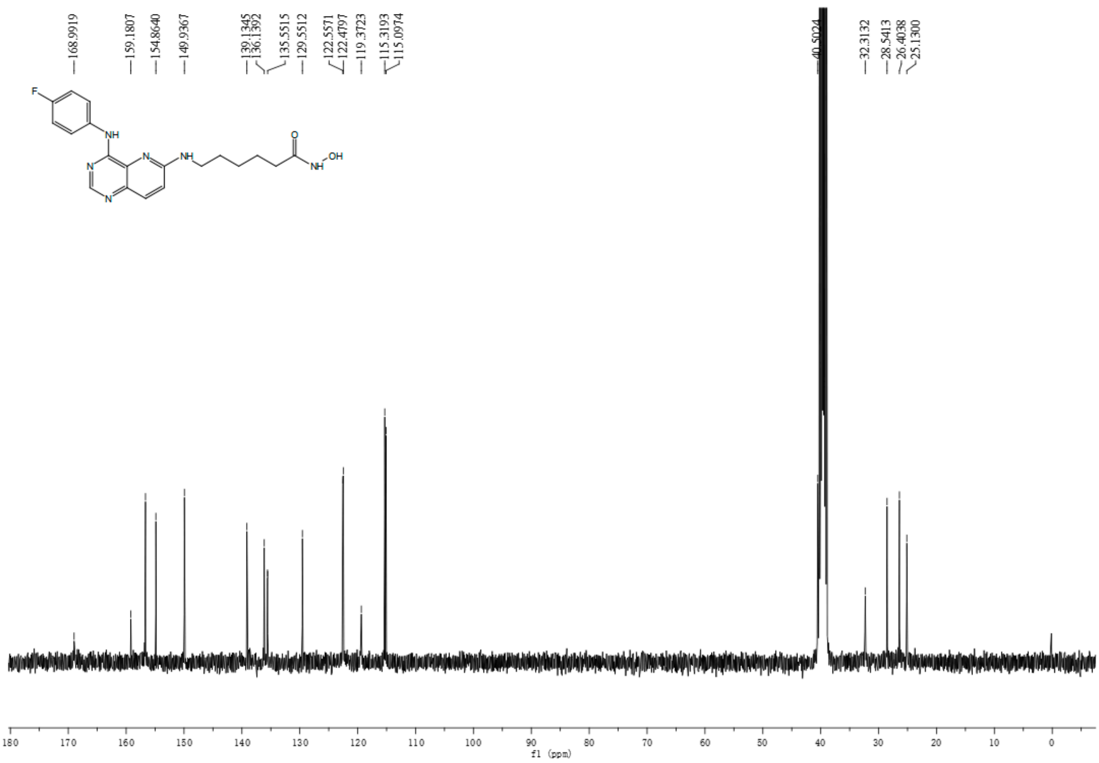

^{13}C-NMR spectra of compound A11

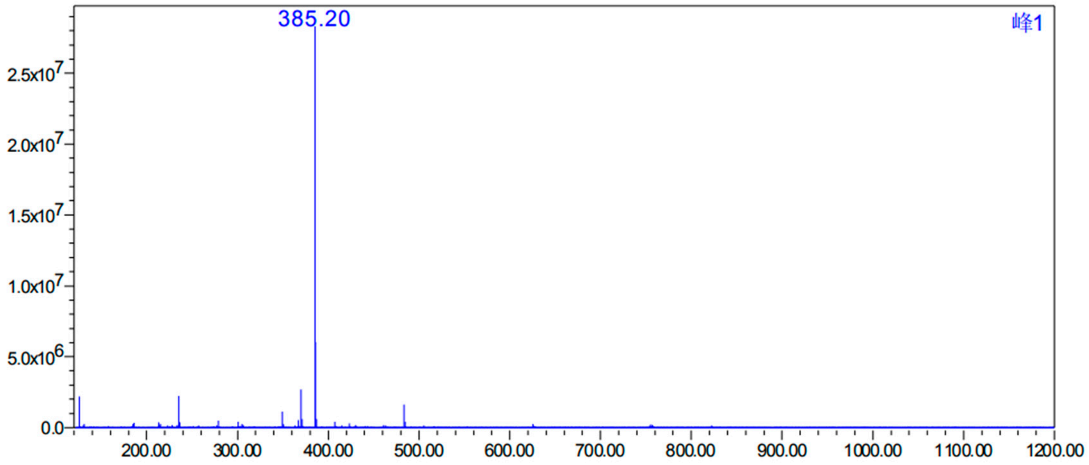

Ms spectra of compound A11

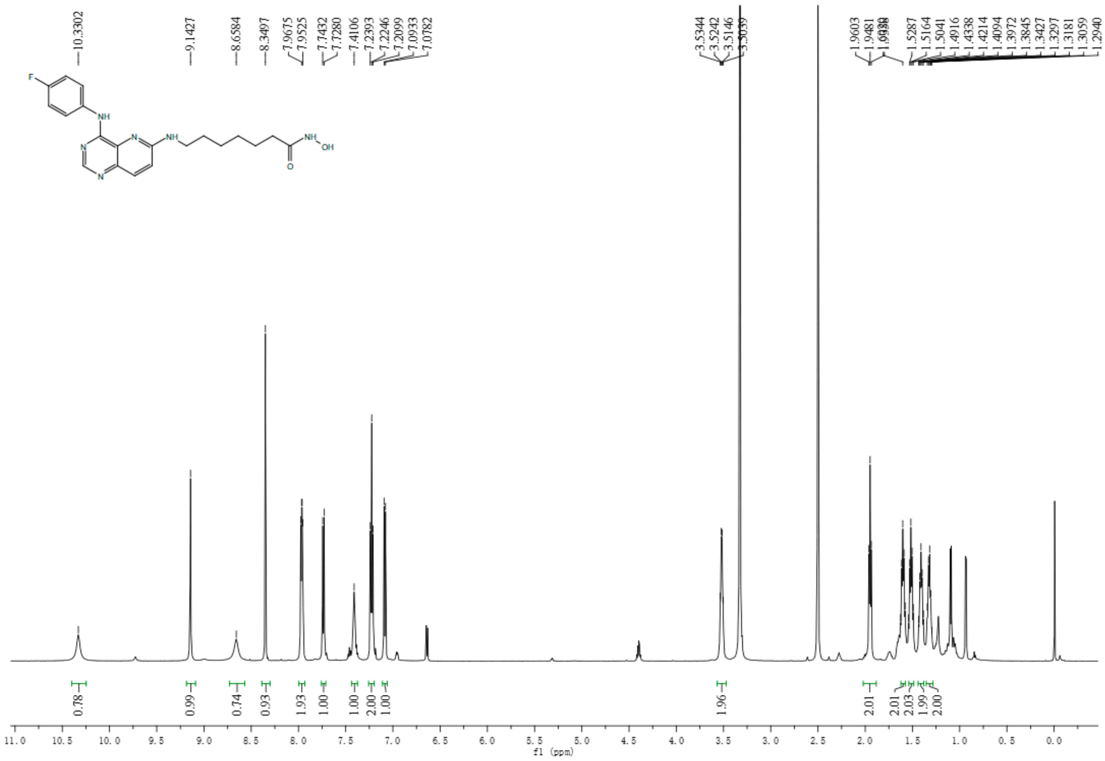

^{1}H-NMR spectra of compound A12

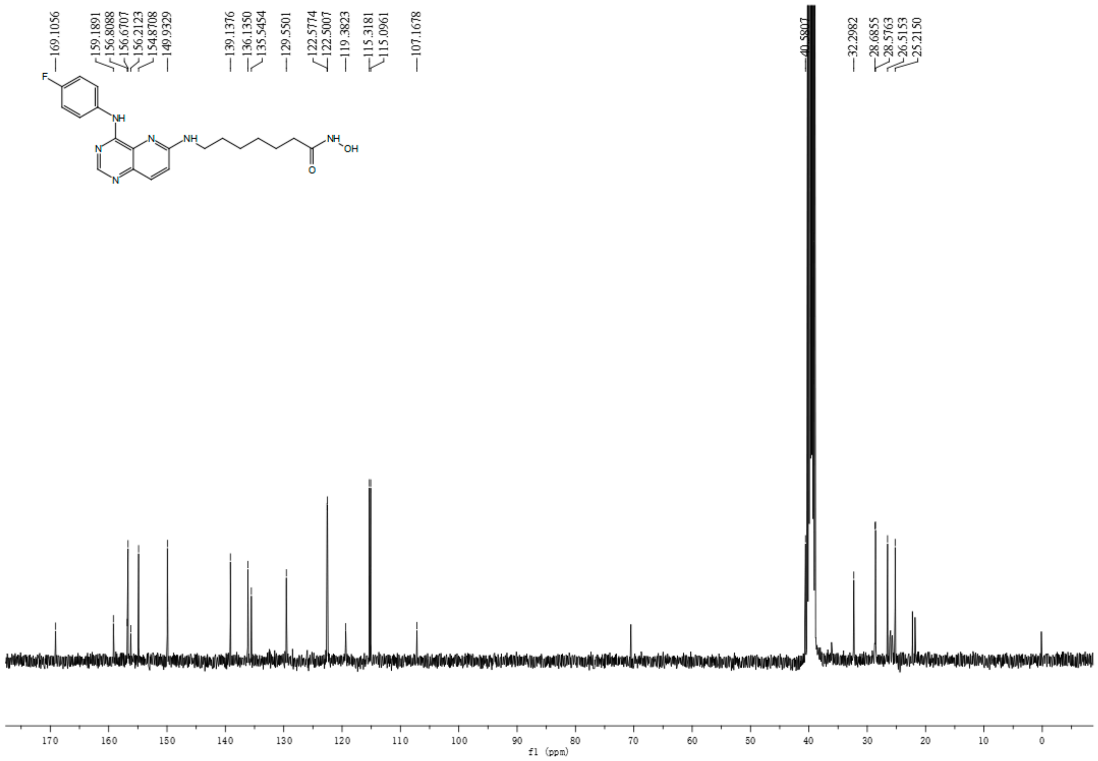

^{13}C-NMR spectra of compound A12

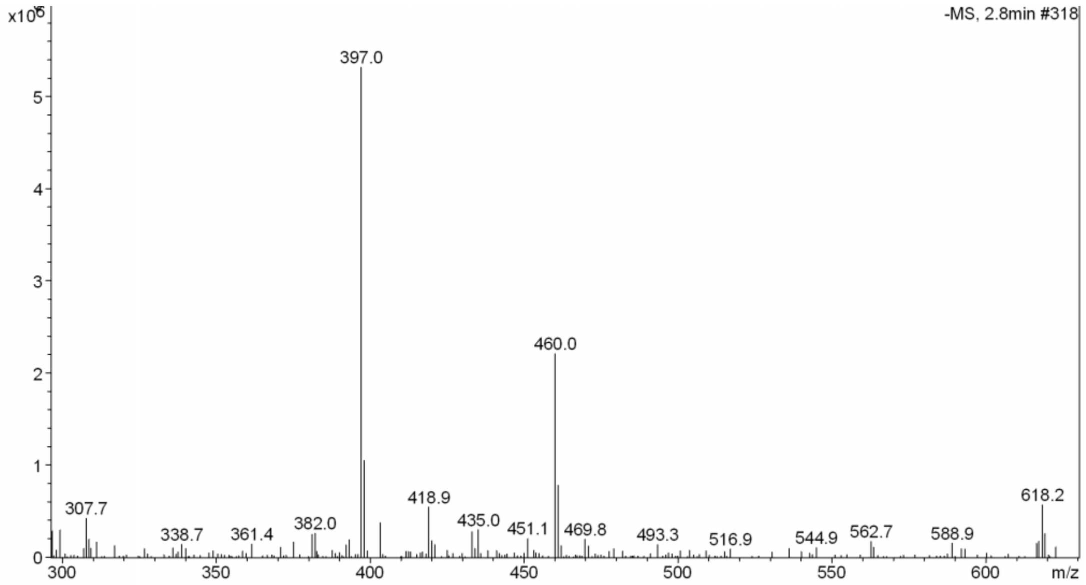

Ms spectra of compound A12

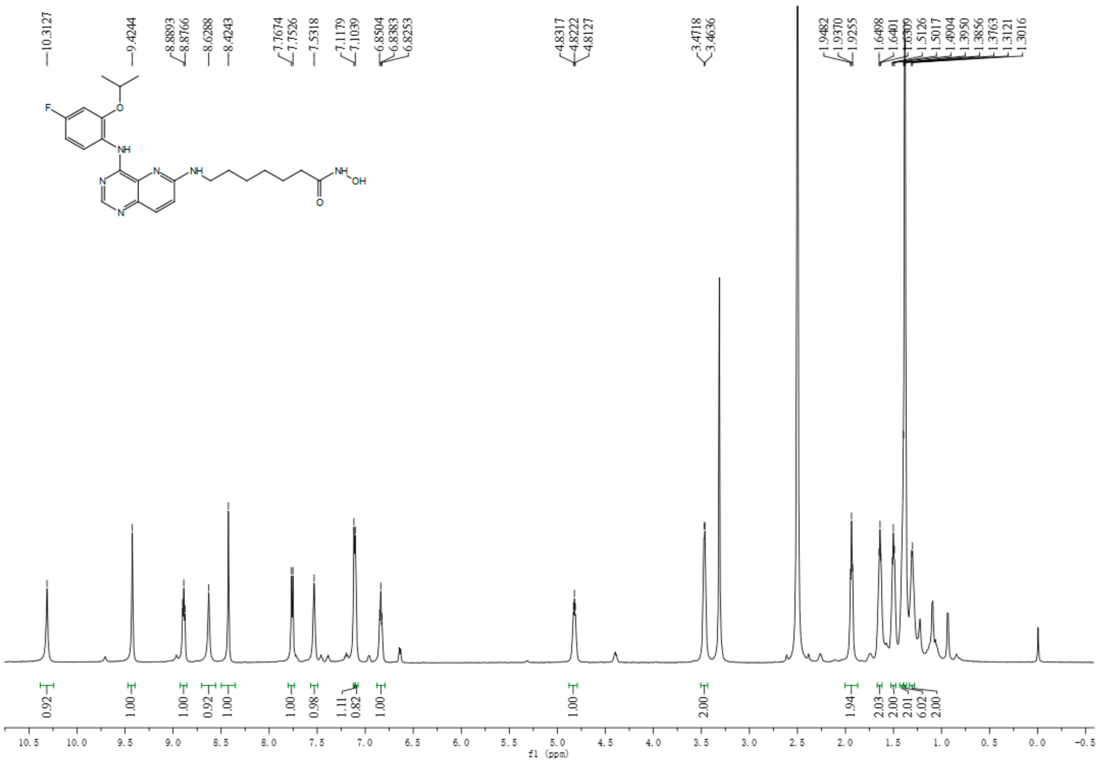

^{1}H-NMR spectra of compound A13

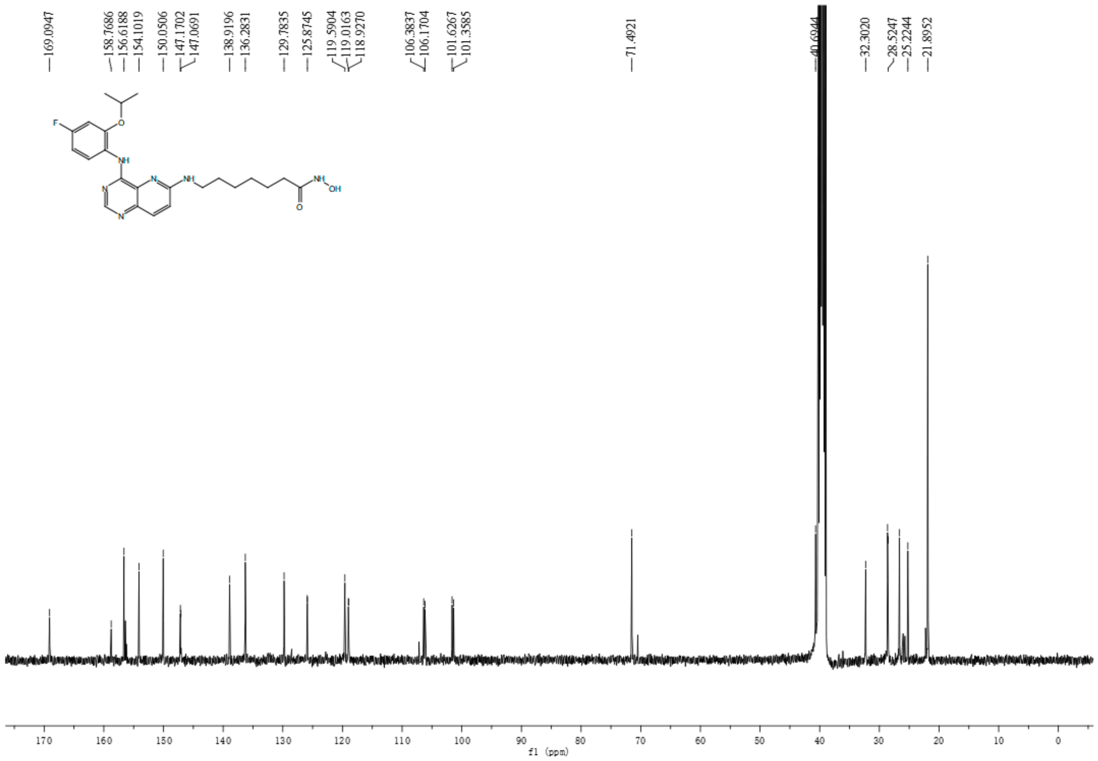

^{13}C-NMR spectra of compound A13

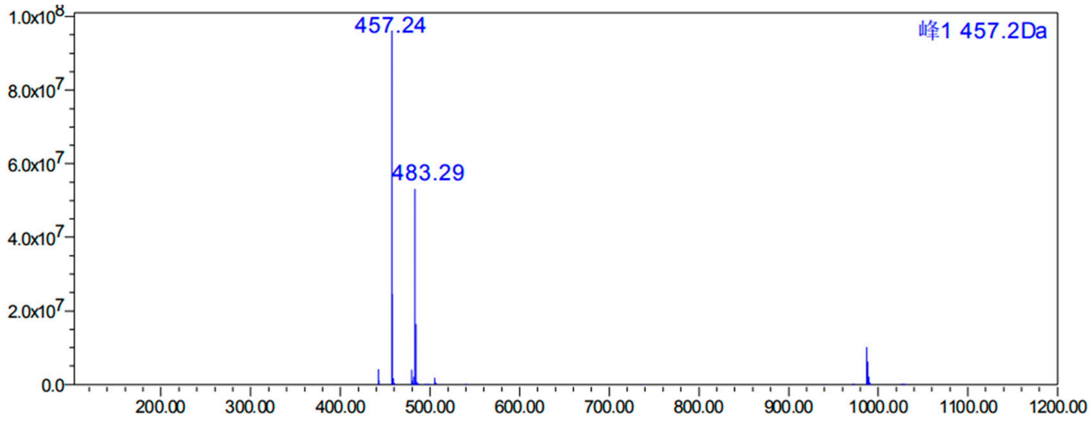

Ms spectra of compound A13

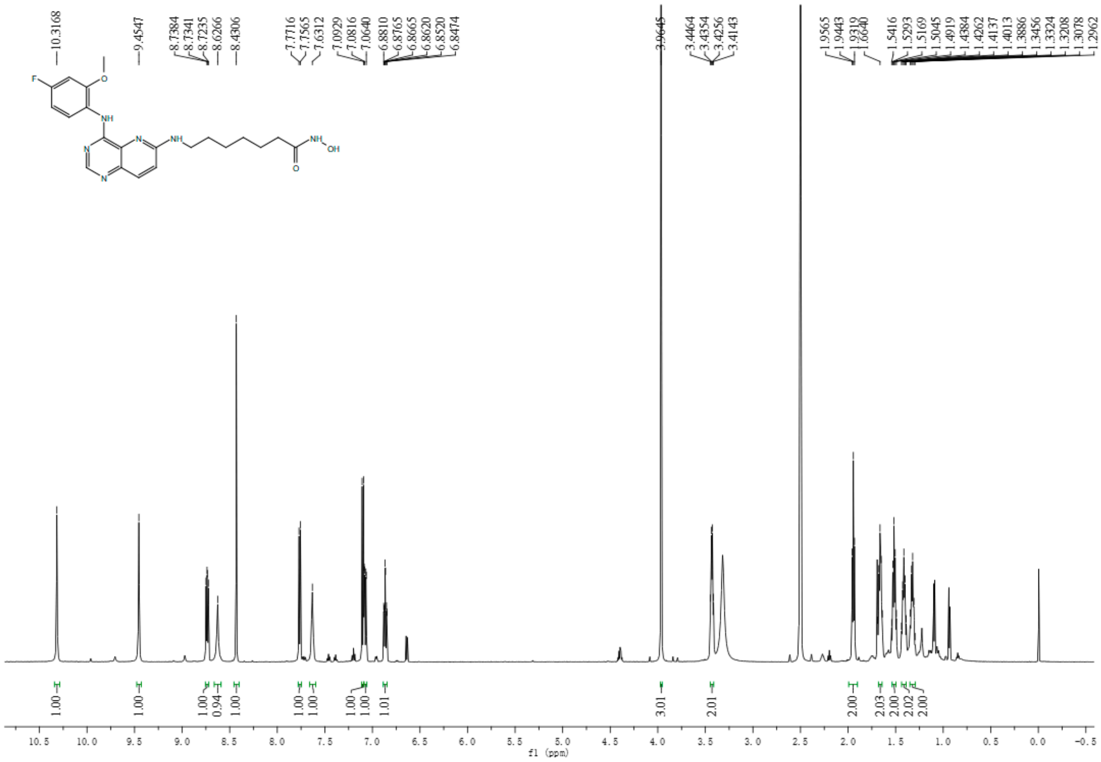

^{1}H-NMR spectra of compound A14

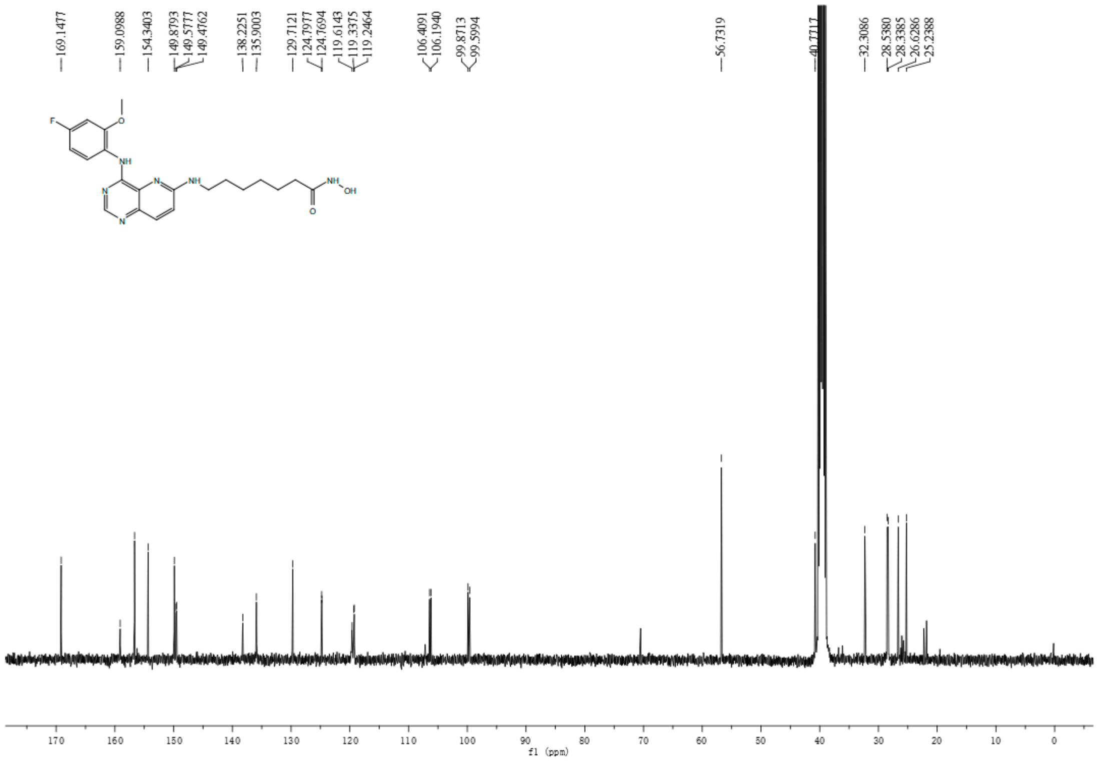

^{13}C-NMR spectra of compound A14

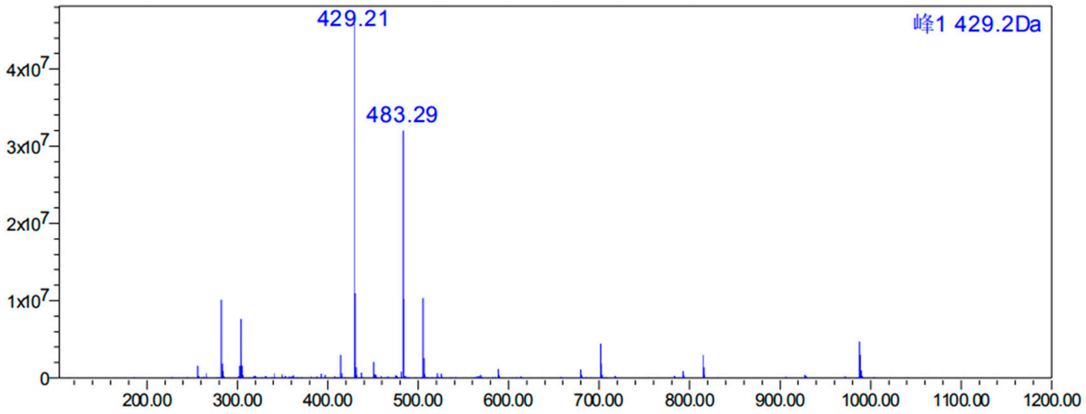

Ms spectra of compound A14

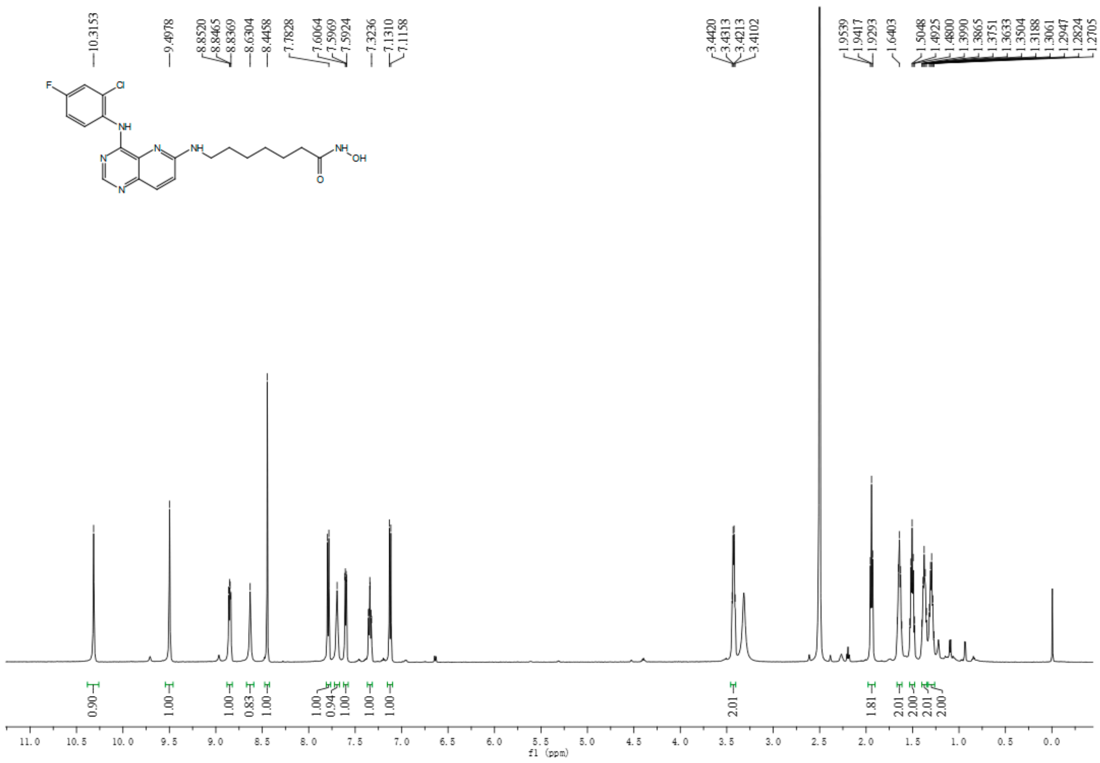

^{1}H-NMR spectra of compound A15

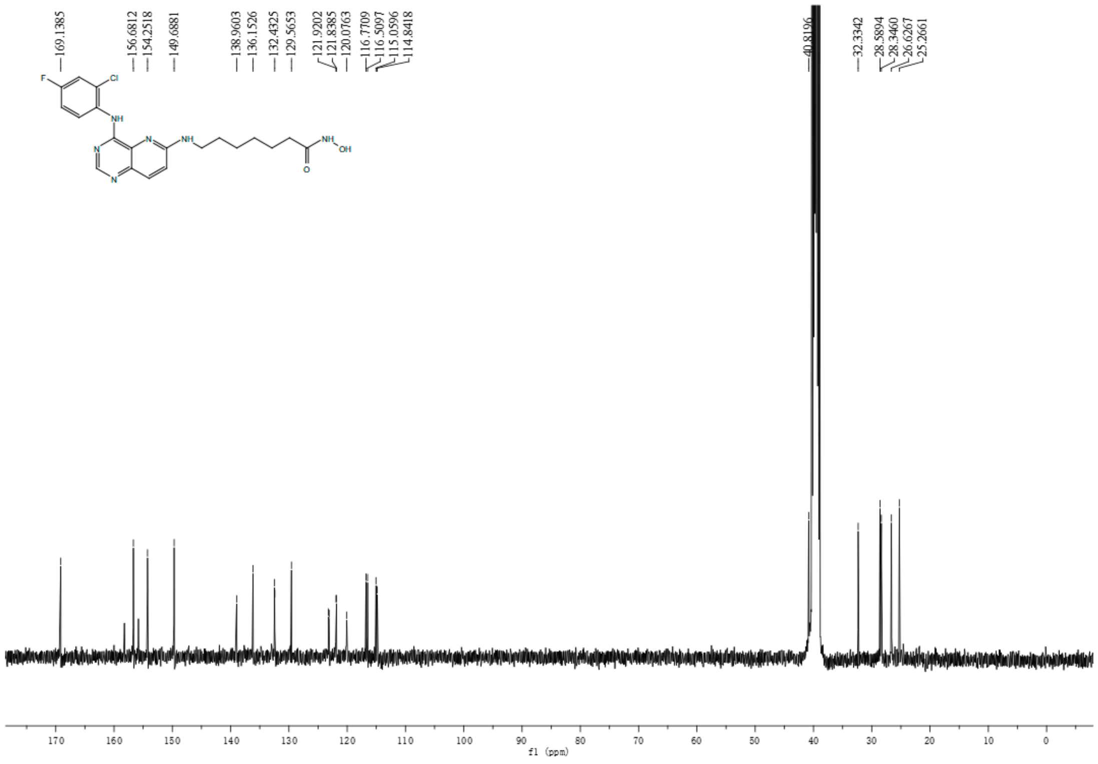

^{13}C-NMR spectra of compound A15

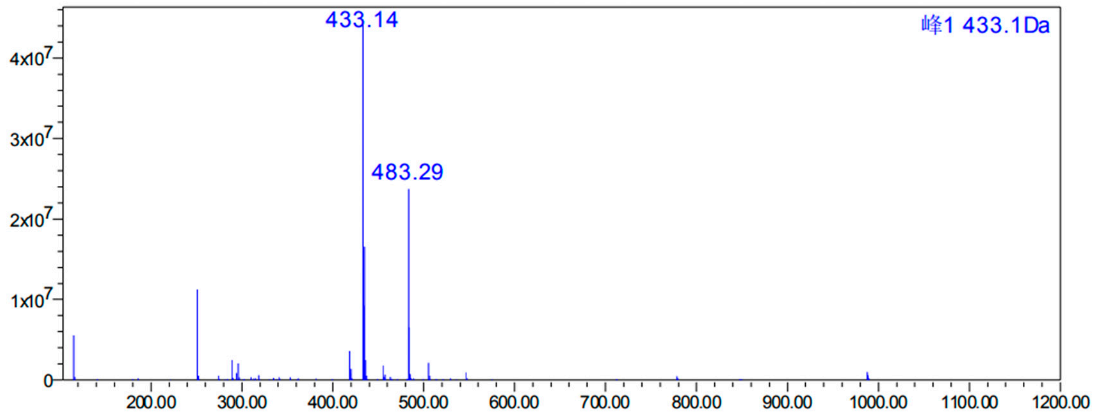

Ms spectra of compound A15

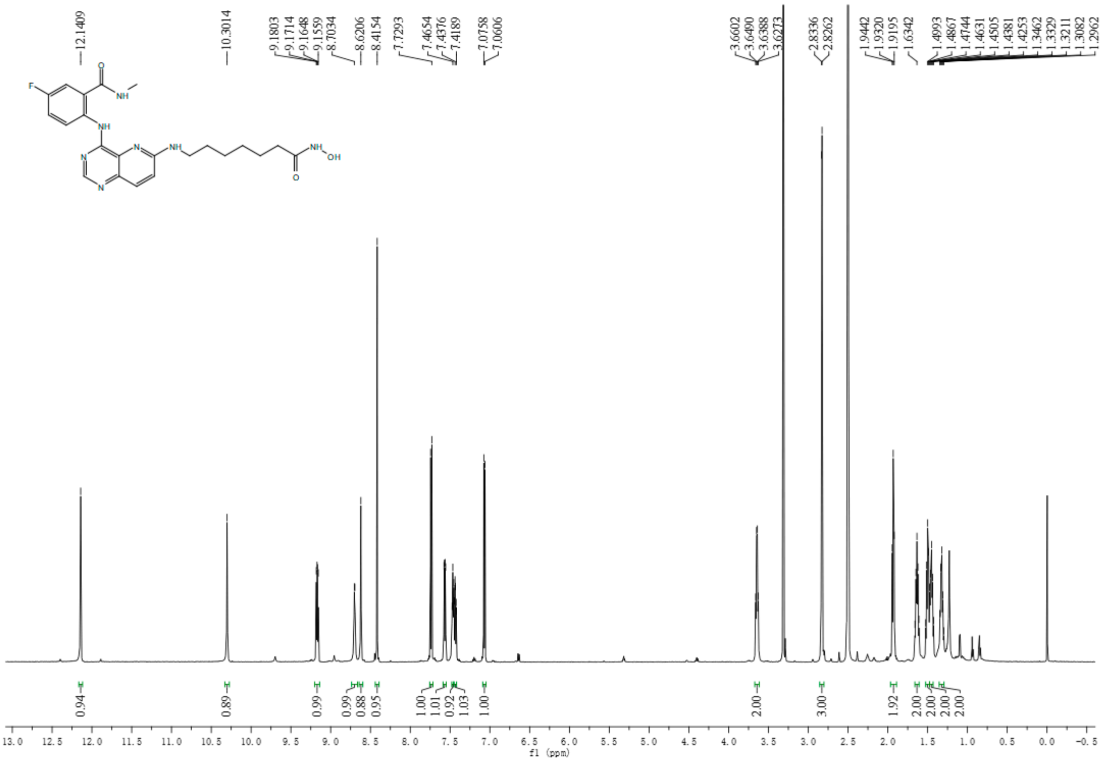

^{1}H-NMR spectra of compound A16

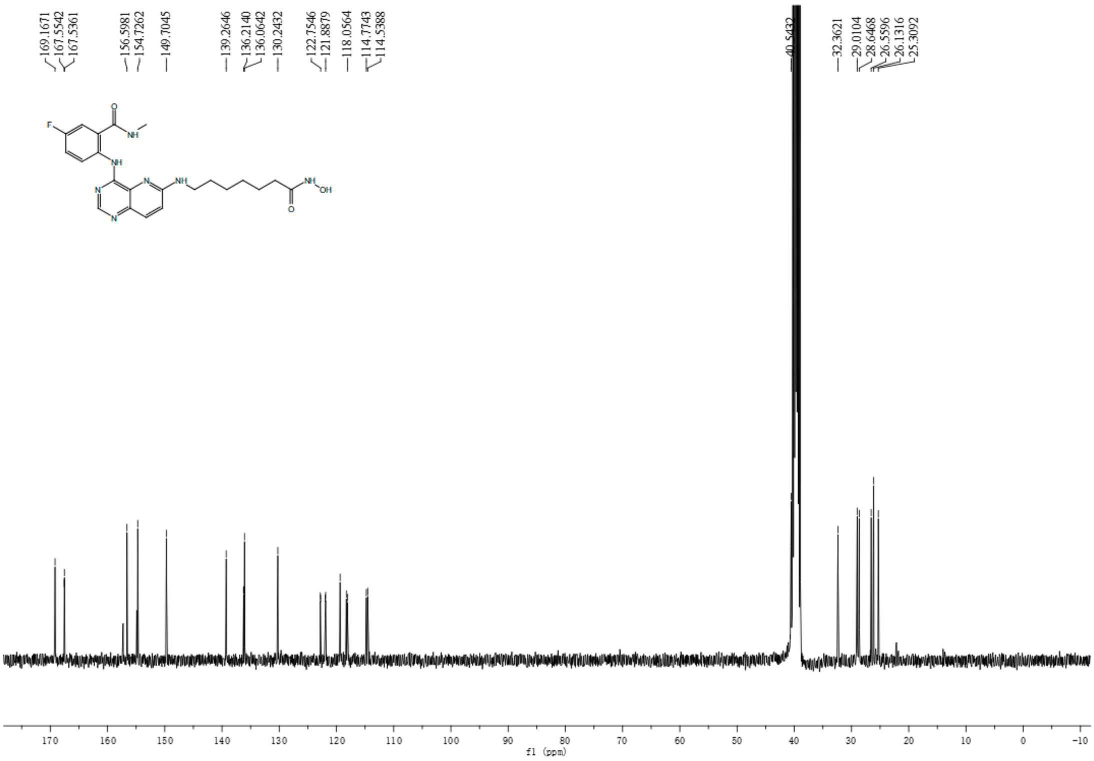

^{13}C-NMR spectra of compound A16

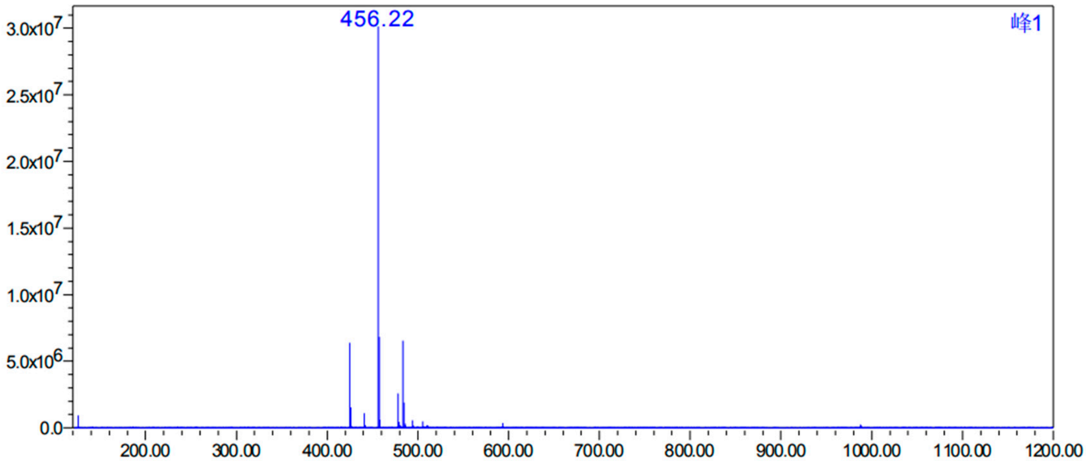

Ms spectra of compound A16

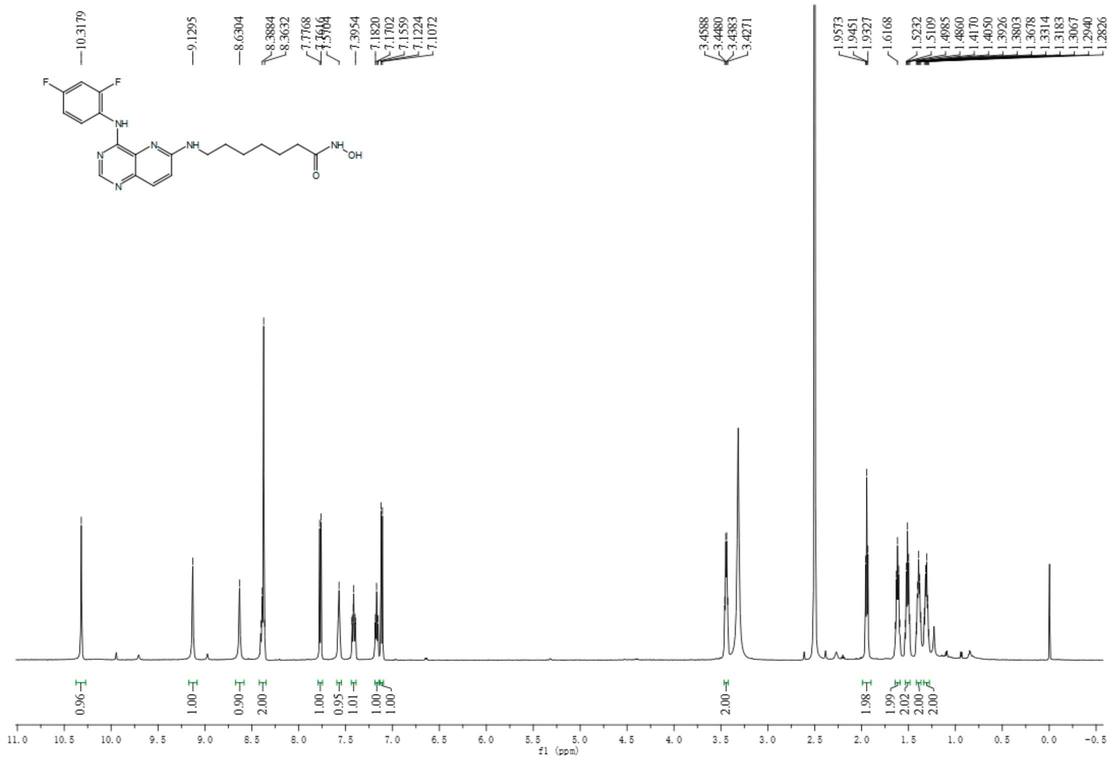

^{1}H-NMR spectra of compound A17

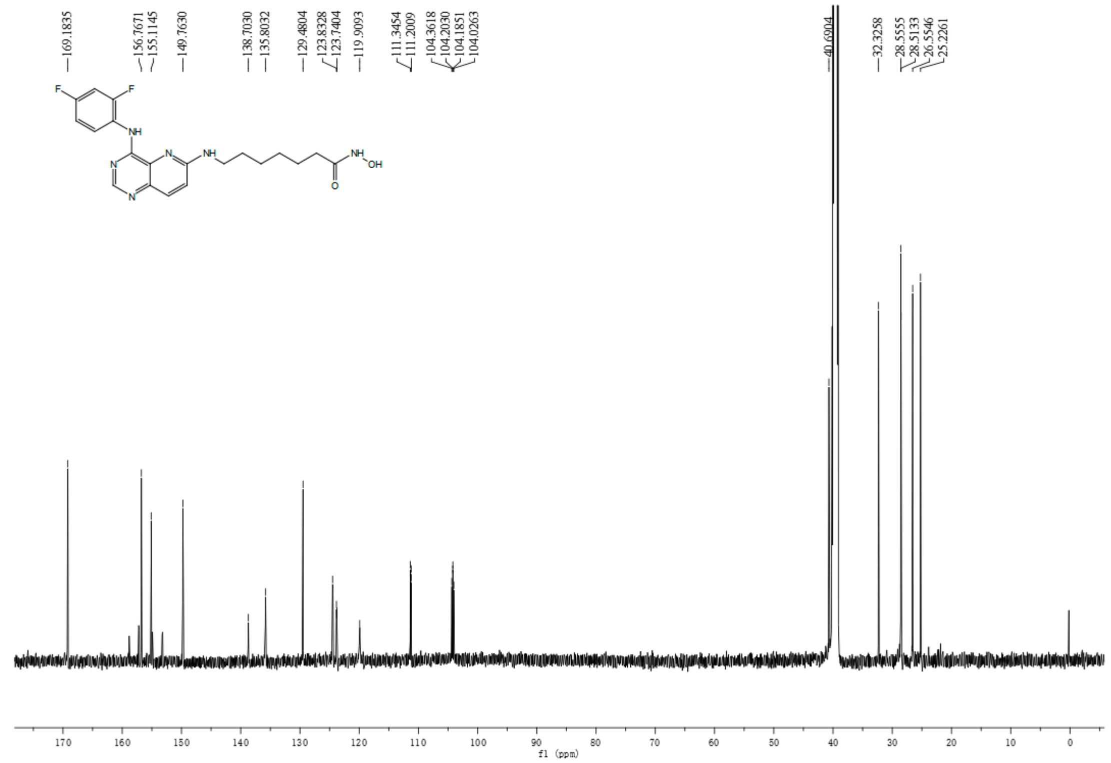

^{13}C-NMR spectra of compound A17

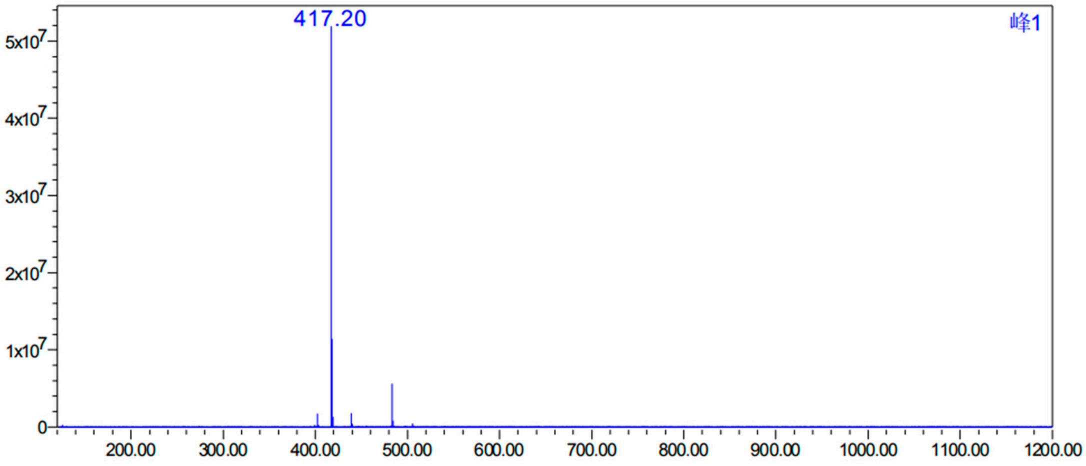

Ms spectra of compound A17

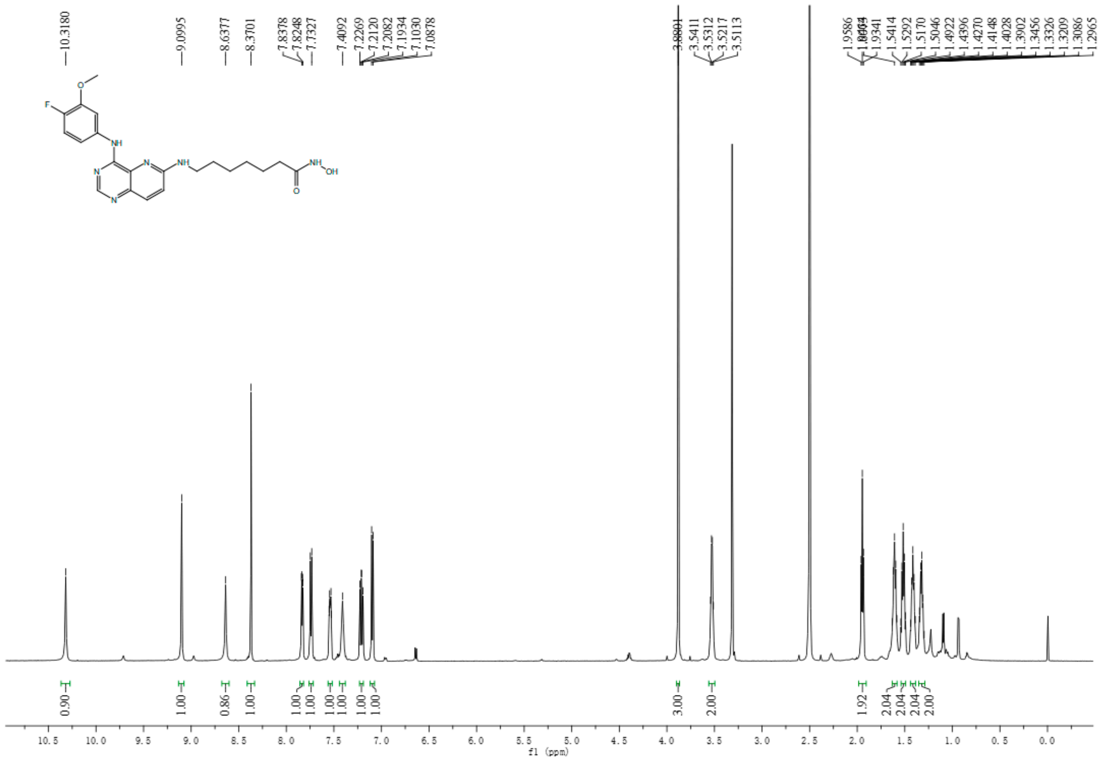

^{1}H-NMR spectra of compound A18

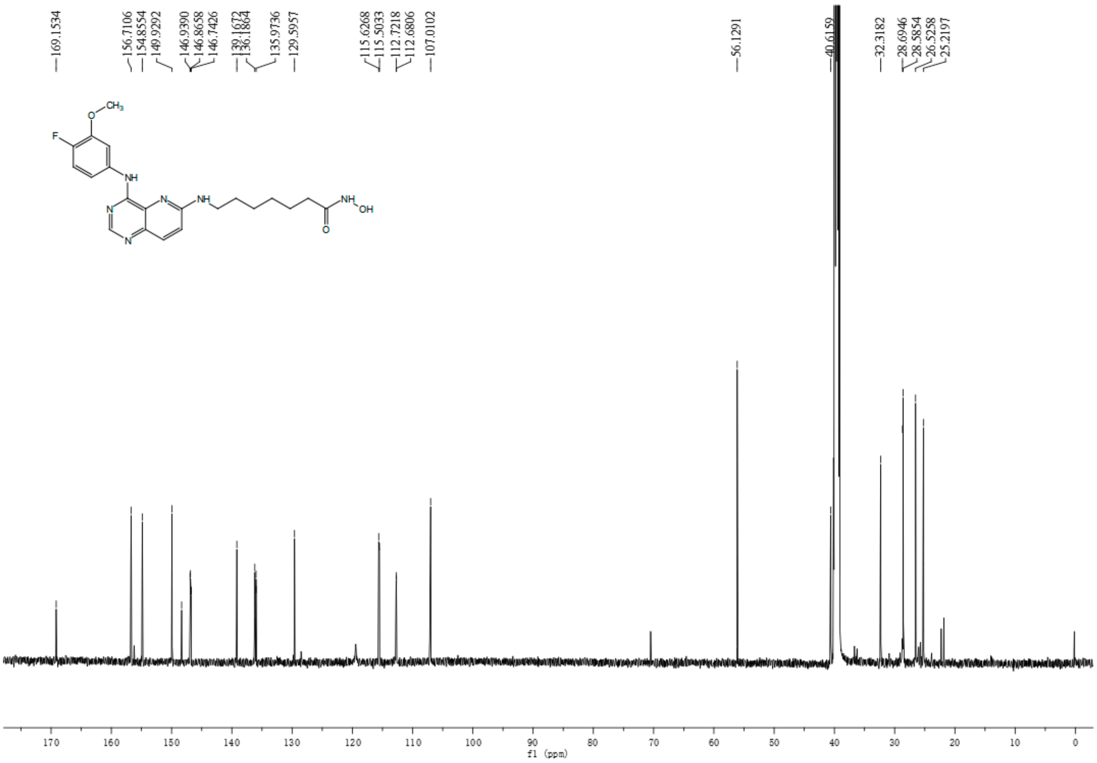

^{13}C-NMR spectra of compound A18

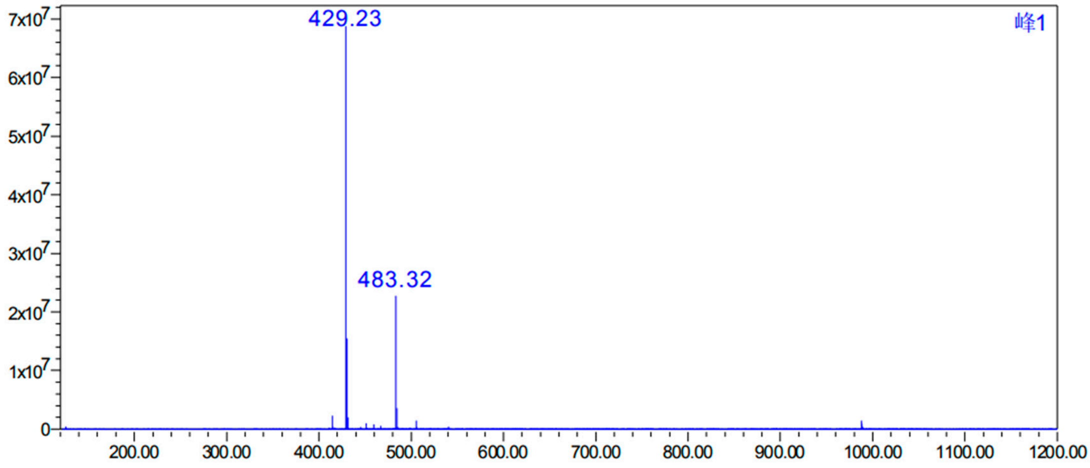

Ms spectra of compound A18

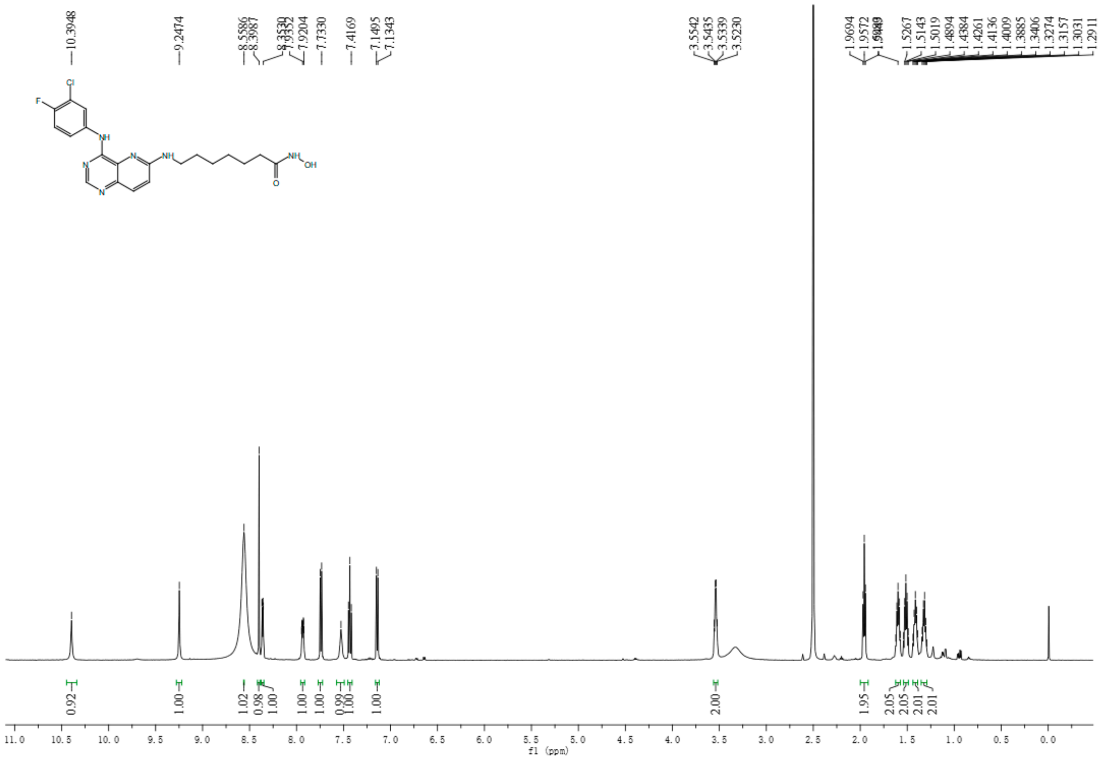

^{1}H-NMR spectra of compound A19

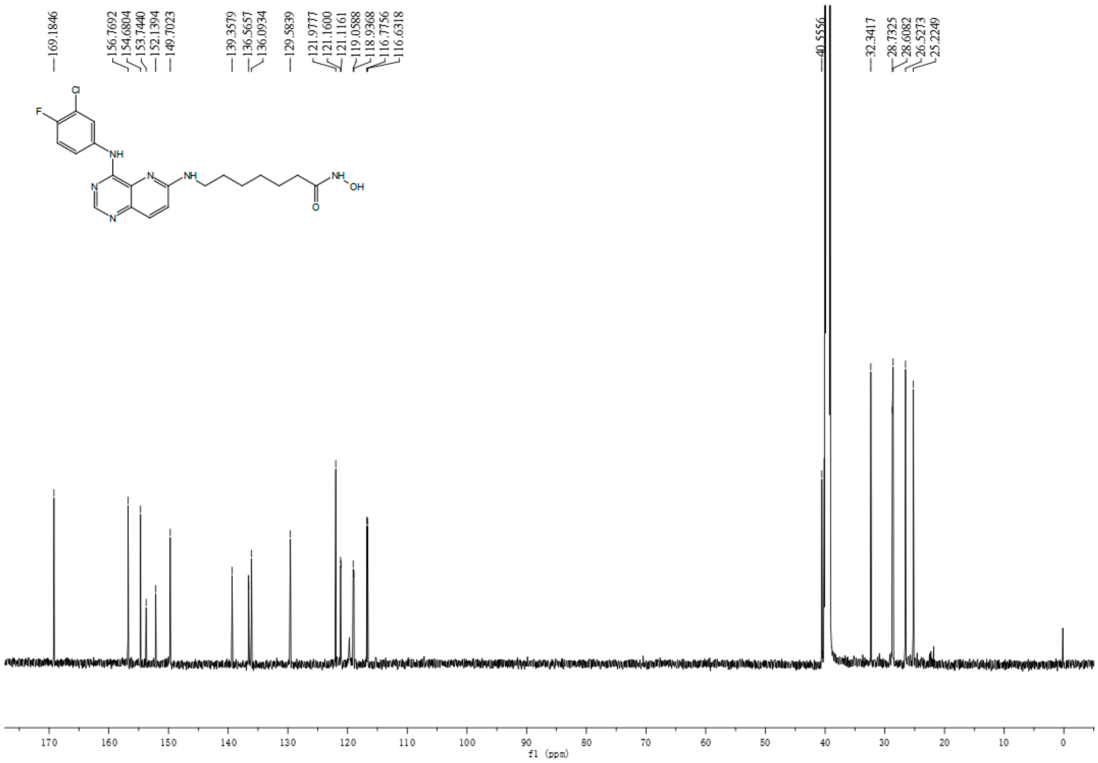

^{13}C-NMR spectra of compound A19

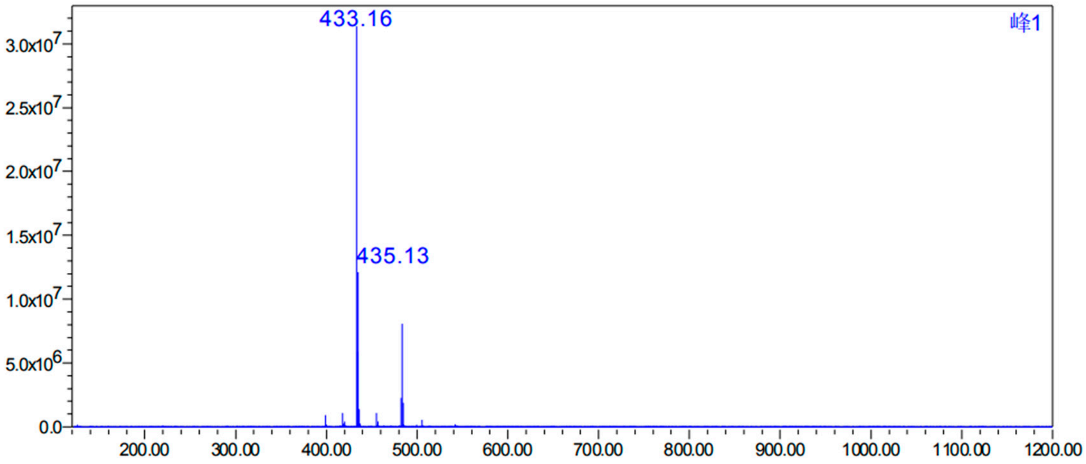

Ms spectra of compound A19
